# Solvation-Driven Self-Assembly of Polyetheramine–Epoxide Gels: Insights from Molecular Simulations

**DOI:** 10.1021/acspolymersau.5c00159

**Published:** 2025-12-30

**Authors:** Renato P. Orenha, Eduardo F. Molina, Felipe B. Alves, Bruno A. Fico, Marcelo Albuquerque, Renato L. T. Parreira, Luciano T. Costa

**Affiliations:** † Núcleo de Pesquisas em Ciências Exatas e Tecnológicas, 92917 Universidade de Franca, 14404-600 Franca, SP, Brazil; ‡ Instituto de Física, 28110 Universidade Federal Fluminense, Av. Gal. Milton Tavares de Souza, 24210-346 Niterói, RJ, Brazil; § MolMod-CS, Instituto de Química, Universidade Federal Fluminense, 24020-150 Outeiro de São João Batista, RJ, Brazil

**Keywords:** polymer-based nanostructures, water solvation, radial distribution function (RDF), solvent accessibility, noncovalent interactions

## Abstract

Polymer-based materials have emerged as promising platforms for controlled nutrient delivery in agriculture, offering enhanced efficiency and sustainability. In this study, we performed molecular dynamics simulations to investigate the self-assembly mechanism of Medipacs epoxy polymer (MEP113) vesicles in aqueous environments. We show that MEP113 concentration critically influences its aggregation behavior: the simulation in the gas phase (vacuum) favors polymer clustering, while aqueous solvation promotes molecular dispersion through hydration effects. Radial distribution function (RDF) analyses revealed that water disrupts direct polymer–polymer interactions, altering the solute organization, especially at higher polymer concentrations. We analyzed structural parameters such as radius of gyration and end-to-end distance measurements and found that solvated systems undergo conformational expansion, contrasting with the compact arrangements observed in dry conditions. Additionally, noncovalent interaction analyses highlighted the key roles of hydrogen bonding and van der Waals forces in stabilizing MEP113 assemblies in solution. These insights advance the rational design of nanostructured polymer systems for applications in agriculture and nanotechnology.

## Graphical abstract

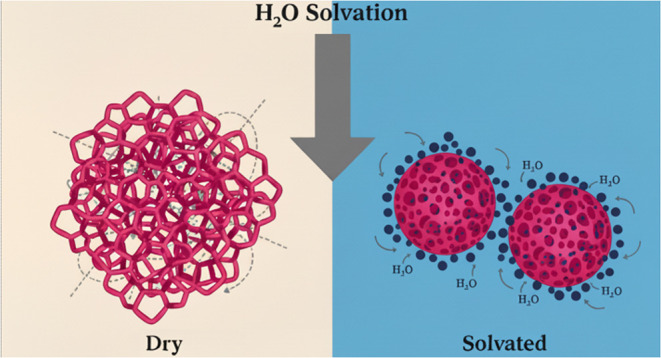

## Introduction

Polymers play a central role in recent decades due to their broad applicability across multiple disciplines, including medicine,^1–3^ engineering,^4^ chemistry,^5^ and, more recently, agronomy. In agriculture, polymers are increasingly being employed to enhance the efficiency and sustainability. For example, superabsorbent hydrogels have been shown to improve soil water retention, reducing irrigation requirements.^6^ Biodegradable polymer coatings slowly release fertilizers and pesticides into the soil, minimizing nutrient loss and reducing environmental impact.^7,8^ Polymeric nanoparticles are also being explored as carriers for agrochemicals, enabling precise delivery to targeted plant tissues.^9,10^ Computational methods have become indispensable for elucidating the structure–property relationships of polymers at the molecular scale.^11–15^ In multiphase systems such as emulsions, simulations have revealed how polymer “brushes” grafted onto particle surfaces can stabilize dispersions by modulating interfacial interactions.^13^

Amine–polyether–epoxide polymers have recently emerged as multifunctional gel systems with significant potential in sustainable agriculture. These materials have been shown to enhance seed germination, nutrient uptake, and early plant growth.^16^ For example, polyetheramine–epoxide nanogels loaded with zinc sources have demonstrated effectiveness as nano-enabled seed treatments, improving germination rates and micronutrient assimilation.^17^ Similar platforms have stimulated seedling vigor and nutrient absorption in crops such as maize and cucumber.^16^ Beyond agriculture, their versatility has been demonstrated in biomedical applications, including the controlled antifungal delivery of amphotericin B via nanogel carriers.^18^ These findings highlight the promise of polyetheramine–epoxide gels as bioactive delivery platforms in both agricultural and medical contexts.^19^ However, despite this progress, no studies to date have applied computational approaches, such as molecular dynamics simulations or density functional theory, to investigate the molecular-level behavior, structure–function relationships, and performance mechanisms of amine–epoxide polymer systems. Herein, we hypothesize that the self-assembly and stabilization of MEP113 gel-forming units emerge from specific hydration patterns and noncovalent interactions that cannot be resolved experimentally. By elucidating these microscopic mechanisms, our simulations aim to establish a clear structure–function relationship linking polymer concentration, solvation, and the formation of vesicle-like assemblies relevant to the performance of these gels. Although classical MD with fixed-charge force fields provides an efficient route to explore these mesoscale processes, it inherently lacks the electronic accuracy of DFT-based descriptions. For this reason, while implicit-solvent models are computationally more affordable, the inclusion of a limited number of explicit water molecules can further refine the representation of key hydration effects without compromising the overall efficiency of the simulations.

Here, the mechanism underlying the formation of an amine–epoxide structure in an aqueous environment has been elucidated using molecular dynamics simulations through structural and energetic analyses. The structural characterization was performed using density maps, radial distribution functions, and molecular flexibility assessments, while the energetic analysis focused on Coulomb and Lennard–Jones interactions to evaluate the stability of the assemblies formed. The fundamental unit of the amine–epoxide, Medipacs epoxy polymer (MEP) with *x* = *y* = 1, and *z* = 3 or MEP113 (Figure 1a), was selected based on the literature.^20^ This simplified structure reflects the lower bound of the manufacturer’s specification, which indicates a distribution of arm lengths in the actual material. For molecular simulations, such a minimal representative model is commonly employed to reduce structural variability while retaining the key reactive and amphiphilic features of the system. In contrast to the Roy et al. study, which focused on the conformational analysis and electronic properties of isolated MEP molecules in vacuum or implicit environments, the present study extends the investigation to explicit aqueous conditions and evaluates the self-assembly behavior of MEP113 into stable vesicular structures. This approach provides molecular-scale insights into how solvation, concentration, and polymer–polymer interactions govern aggregate formation, information on relevance not only for agricultural gels but also for the broader polymer science community interested in hydration-driven organization and stimuli-responsive materials. To investigate the influence of increasing the number of MEP113 units and the role of solvation in vesicle formation, we explored the following systems (along with the data plot coloring): (**A**) MEP113 (blue); (**B**) 2 MEP113 (green); (**C**) 10 MEP113 (violet); (**A**_**solvated**_) MEP113 + 5,000 H_2_O (red); (**B**_**solvated**_) 2 MEP113 + 5,000 H_2_O (yellow); and (**C**_**solvated**_) 10 MEP113 + 15,000 H_2_O (orange) (Figure 1b). Additionally, to examine potential interactions between vesicles in aqueous solution, a larger system containing two vesicles, (**D**_**solvated**_) 20 MEP113 + 30,000 H_2_O (gray), was investigated (Figure 1c).

**Figure 1:**
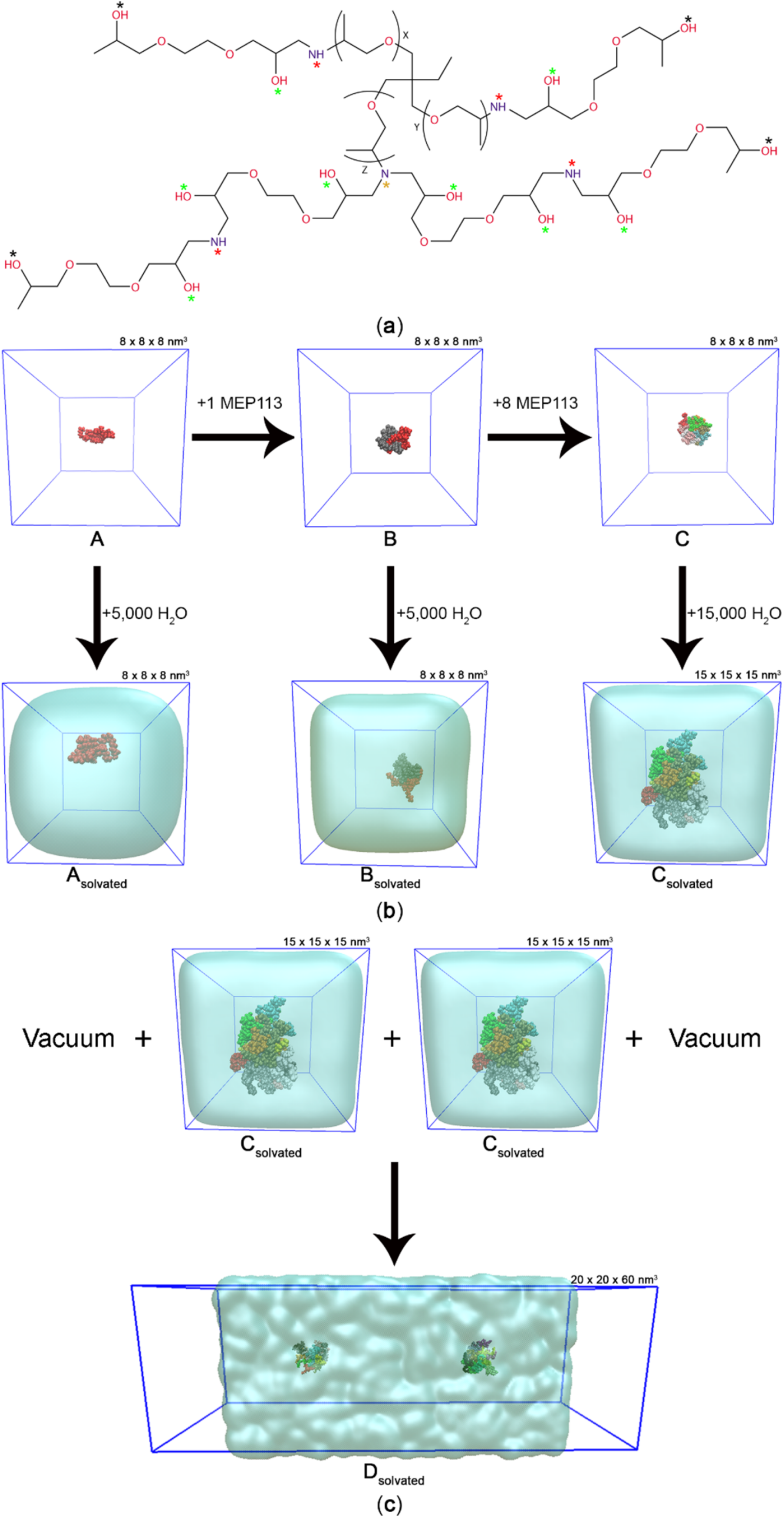
(a) Chemical structure of MEP113 (*x* = *y* = 1, *z* = 3); (b) a schematic representation of simulated systems showing (i) the effect of increasing the number of MEP113 units (**A** → **B** → **C**) and (ii) the role of water solvation (**A**_**solvated**_–**C**_**solvated**_); (c) formation of a two-vesicle system in aqueous medium (**D**_**solvated**_: 20 MEP113 + 30,000 H_2_O). Asterisks indicate functional groups analyzed: central nitrogen (yellow), middle hydroxyl (green), middle amine (red), and terminal hydroxyl (black).

## Results and Discussion

### Validation

We validated the systems by visualizing the decrease of the potential energy per molecule to a minimum value during the geometry optimization performed through the conjugate gradient (CG) method (Figure S4). Next, we obtained parameters such as kinetic, potential, and total energy per molecule, as well as density (for solvated systems only). These parameters remained nearly constant during the final equilibration (Figures S5–S11).

### Structural Parameters

To elucidate the structural properties of the MEP113 systems, we first analyzed the 2D density map of the MEP113 structure (Figure 2a). We observed that increasing the number of MEP113 units (**A** → **B** → **C**) directly increases the concentration of MEP113 molecules in the gas phase (left-hand side). Conversely, the introduction of water into the systems (**A** → **A**_**solvated**_, **B** → **B**_**solvated**_, and **C** → **C**_**solvated**_) leads to a dilution effect, decreasing the MEP113 concentration and influencing the spatial distribution of the polymer in solution. In the **D**_**solvated**_ system, which represents two distinct polymer aggregates arranged in a slab configuration, the 2D density map reveals that the aggregates remained stable and isolated throughout the simulation, and we did not observe significant coalescence between them. The average distance between the centers of mass of the vesicles was 131 ± 11 nm, further supporting the observation that under the simulated conditions, the solvation effect was sufficient to maintain the spatial separation of the polymer assemblies. These findings align well with the experimental results from transmission electron microscopy (TEM) images of the epoxide–amine gels. The gels were formed using synthesis conditions adapted from the literature. (For details on the preparation and TEM characterization of the gels, please refer to the Supporting Information.) Figure 2b shows a micrograph of the epoxide–amine colloidal system, which displays well-defined spherical particles with porosity characteristics. Although the individual subparticles appear spherical in TEM images, the elongated features observed in the 2D density maps can be attributed to the projection of a dynamic three-dimensional distribution, in which solvent-induced density fluctuations and internal structural rearrangements produce apparent anisotropic regions upon averaging; moreover, the different colors in the density map (Figure 2a) illustrate how frequently the subparticles visit specific regions of the simulation box, with the most intense areas (hotter colors) showing an approximately circular shape, similar to that in Figure 2b, whose scale is 1 order of magnitude larger than the simulation box used in our simulations. These features could enable the formation of polymeric colloidal systems known as micro- or nanogels, which allow to retain a large amount of water into the structure.^21^ To our knowledge, this is the first atomistic visualization of hydration-driven organization in amine–epoxide gel units, providing a direct molecular interpretation for experimentally observed porosity and swelling capacity.

**Figure 2:**
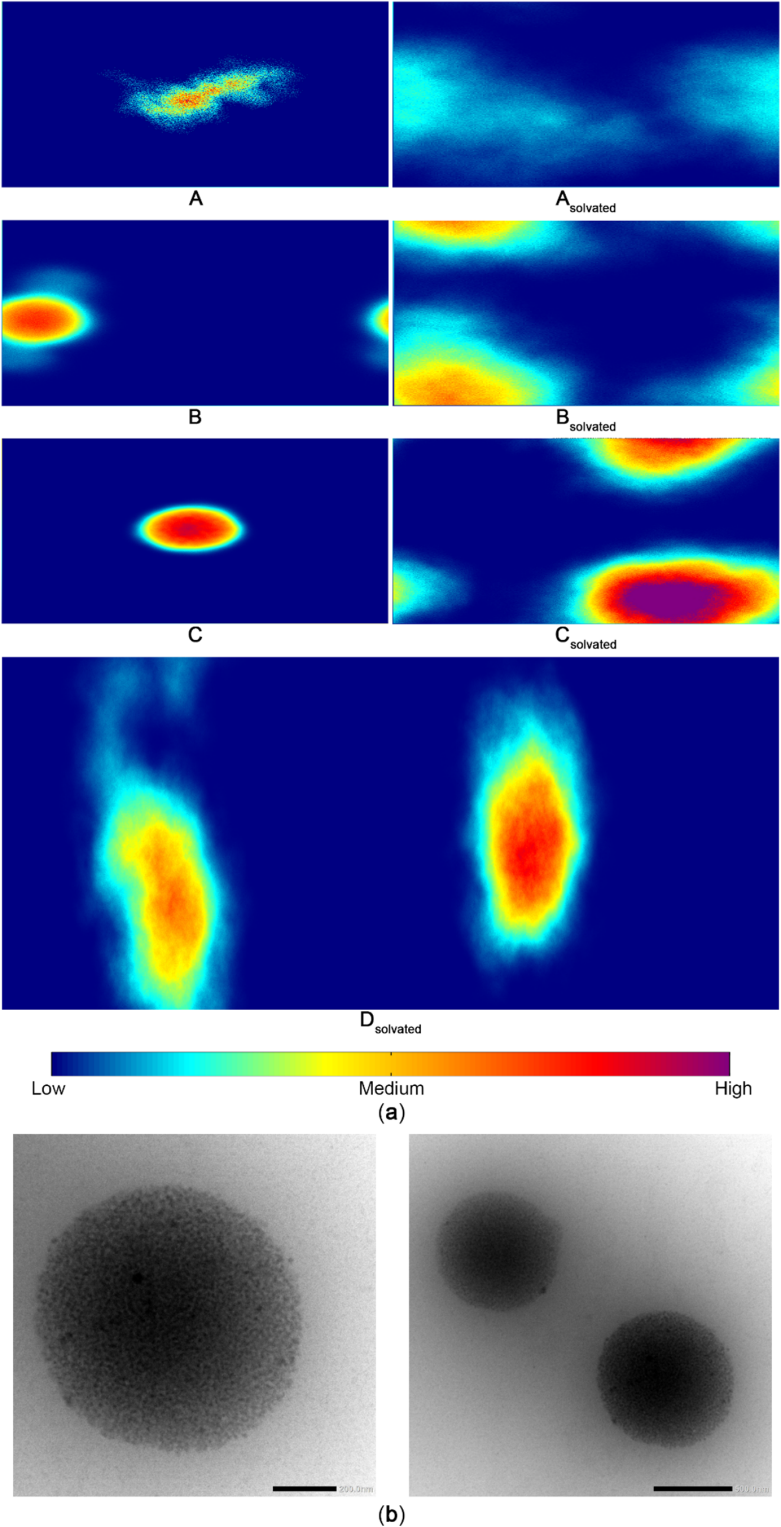
(a) 2D density maps of MEP113 distribution in dry (**A–C**) and solvated systems (**A**_**solvated**_–**D**_**solvated**_), illustrating the effect of polymer concentration and water solvation on molecular organization; (b) TEM micrograph of epoxide–amine colloidal particles synthesized under conditions described in the Supporting Information, showing spherical morphology with porosity features. Scale bars: 200 (left) and 500 nm (right).

Our RDF analysis of the centers of mass of MEP113 reveals important trends regarding MEP113 interactions (Figure 3). In non-solvated conditions, system **B** (2 MEP113) exhibits sharp RDF peaks, indicating preferential intermolecular distances, while system **C** (10 MEP113) displays broader peaks, reflecting the increased molecular clustering. Upon solvation, the RDF peaks diminish in intensity, suggesting that water molecules mediate and intersect directly MEP113 interactions. This effect is most prominent in **C**_**solvated**_, where a higher solvent content fosters a more homogeneous molecular dispersion. Thus, increasing MEP113 concentration enhances direct intermolecular interactions, whereas solvation disrupts these contacts, leading to a more dispersed molecular arrangement. In the **D**_**solvated**_ system, the RDF was calculated exclusively between the centers of mass of the MEP113 units within a single polymer vesicle. The resulting profile reveals persistent intermolecular peaks, indicating that despite the presence of solvent, the polymer chains within each vesicle remain in proximity, preserving a clustered organization. These results confirm that at high local concentrations, the polymer–polymer interactions within an aggregate are strong enough to sustain a compact structure, while solvation prevents coalescence between separate vesicles, as evidenced by the structural stability observed in the density maps. This concentration-dependent transition from compact to solvent-stabilized conformations represents a previously undescribed structural regime for amine–epoxide gels, revealing how aggregation scales with monomer number and hydration. These unprecedented findings on epoxide–amine polymeric gels provide a fundamental basis for elucidating the physicochemical mechanisms that regulate nutrient release and mediate the interactions of these colloidal matrices with micro- and macronutrients. Such insights not only advance the molecular-level understanding of gel–nutrient dynamics but also highlight the potential of these systems as innovative platforms for the development of sustainable agricultural technologies. Since this class of polymer was recently applied as biomaterials for seed priming applications,^16^ this work using theoretical simulation unveils important solvation processes of the epoxide–amine colloids for application as swollen networks in sustainable agriculture.

**Figure 3:**
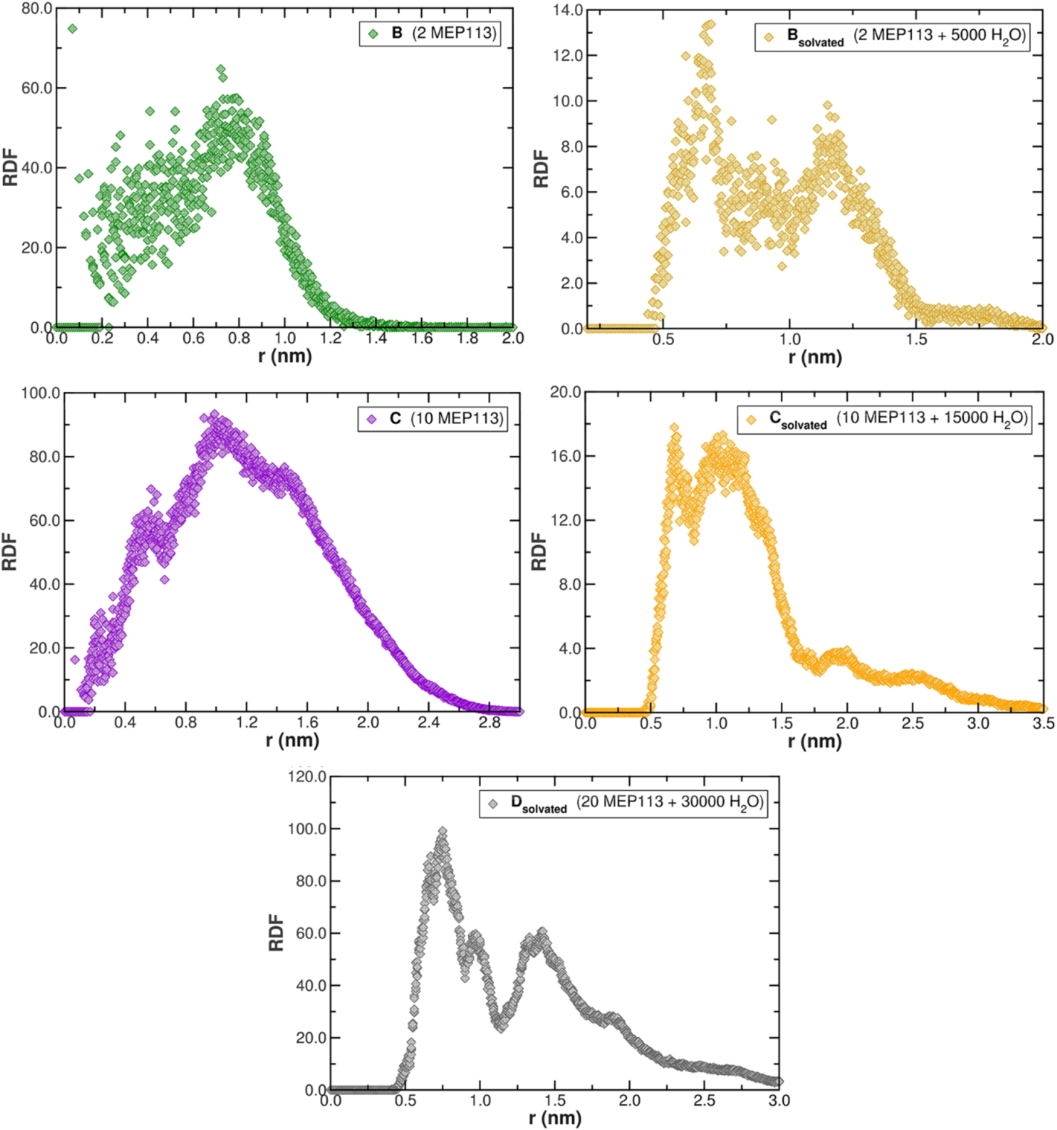
Radial distribution functions (RDF) between centers of mass of MEP113 units in dry systems (**B**, **C**) and solvated systems (**B**_**solvated**_–**D**_**solvated**_). For **D**_**solvated**_, the RDF was calculated within only a single vesicle. The comparison highlights solvent-mediated disruption of polymer–polymer contacts and preservation of intravesicular clustering.

Further RDF analysis explores the hydration structure by considering interactions between MEP113 and water as well as water–water organization (Figure S12). In systems with low polymer concentration–MEP113 molecules (**A**_**solvated**_ and **B**_**solvated**_), the polymer–water RDF stabilizes around 1 nm, indicating a well-defined hydration shell. In contrast, the hydration of **C**_**solvated**_ is more complex, displaying a slower RDF plateau formation due to a higher polymer concentration. The water–water RDFs (Figure S12) display a characteristic peak at ∼0.3 nm, indicative of a structured hydrogen-bond network, which remains essentially unchanged across different MEP113 concentrations. This suggests that despite the increasing presence of MEP113, the bulk water structure is largely preserved, maintaining its inherent hydrogen-bond organization even in more concentrated polymer solutions. In **D**_**solvated**_, the RDF analysis indicates that the two polymer aggregates within the slab remain spatially separated throughout the simulation, as evidenced by the similar polymer–water and water–water RDF profiles to those of **C**_**solvated**_, suggesting stable isolation of the polymer clusters in the solvated environment. These features agree with the literature where these amine–epoxide particles showed a high amount of water content to stabilize as a spherical morphology.^21^ Such consistency highlights the robustness of the computational model, which not only captures the essential structural features observed experimentally but also provides molecular-level insights into the solvation-driven stabilization of these complex systems. This agreement underscores the model’s ability to bridge simulation and experiment, strengthening confidence in its predictive power.

The molecular flexibility of MEP113 was examined through the average values of the radius of gyration (*R*_g_) and the end-to-end distance (*d*_end‑to‑end_), obtained from the histograms presented in Figures S13 and S14 during the final 15 ns of NPT equilibration at 300 K. In dry conditions, system **A** (1 MEP113) presents the largest *R*_g_ (1.045 nm) and *d*_end‑to‑end_ (2.883 nm), indicative of an extended conformation. In contrast, system **B** (2 MEP113) exhibits a more compact structure, with *R*_g_ and *d*_end‑to‑end_ values of 0.793 and 1.400 nm, respectively. System **C** (10 MEP113) shows intermediate values (*R*_g_ = 0.879 nm, *d*_end‑to‑end_ = 1.708 nm), suggesting molecular aggregation that limits individual chain extension.

Upon solvation, a distinct trend emerges: **A**_**solvated**_ (1 MEP113 + 5000 H_2_O) undergoes compaction (*R*_g_ = 0.816 nm, *d*_end‑to‑end_ = 1.643 nm), reflecting the polymer’s collapse in the aqueous environment. Conversely, systems **B**_**solvated**_ and **C**_**solvated**_ display increased *R*_g_ (0.922 and 0.962 nm) and *d*_end‑to‑end_ values (2.077 and 2.227 nm, respectively), indicating a transition to more extended conformations due to water-mediated stabilization and reduced polymer–polymer interactions.

In the **D**_**solvated**_ system, which contains two polymer vesicles, the average *R*_g_ (0.920 nm) and end-to-end distance (1.771 nm) indicate a moderately compact structure, comparable to that of **B**_**solvated**_ and slightly more contracted than that of **C**_**solvated**_. This suggests that even within densely packed aggregates, individual polymer chains can adopt semi-extended conformations stabilized by solvation, while the overall aggregate remains structurally cohesive. This characteristic could lead to the formation of micro- and nanogels since the number of monomers to produce amine–epoxide colloids can determine the final size of the particles and properties. A summary of these results is presented in Table S1 and Figures S13 and S14.

These findings support the 2D density maps and RDF analyses, emphasizing that dry systems tend to aggregate into compact structures, whereas solvated systems promote polymer expansion and structural rearrangement due to hydration effects.

The SASA) of each MEP113 unit was evaluated in both **C**_**solvated**_ and **D**_**solvated**_ systems to complement the structural parameter analysis. The average values were 10 ± 2 nm^2^ and 12 ± 3 nm^2^, respectively (Figure 4). In the **C**_**solvated**_ system, the SASA values indicate a relatively high solvent exposure for each polymer chain, reflecting the stabilization provided by the hydration shell and moderate polymer–polymer interactions within the single vesicle. Interestingly, in the **D**_**solvated**_ system, where two vesicles coexist within the same simulation box, the average SASA per polymer chain, calculated for one of the vesicles, is higher than in the **C**_**solvated**_ system. This increase suggests that the proximity of another vesicle in the same solvated environment promotes a slight loosening or reorganization of the polymer chains within each vesicle, enhancing the solvent exposure of some chains. This behavior indicates that the presence of a second vesicle may locally perturb the folding of polymer chains within the neighboring vesicle, increasing the accessible surface area without causing coalescence or compromising the structural integrity of the assemblies. These findings are consistent with the trends observed in the 2D density maps and RDF results, confirming that the vesicles remain spatially separated but experience subtle intervesicular effects that modulate the organization and solvation of individual polymer chains.

**Figure 4:**
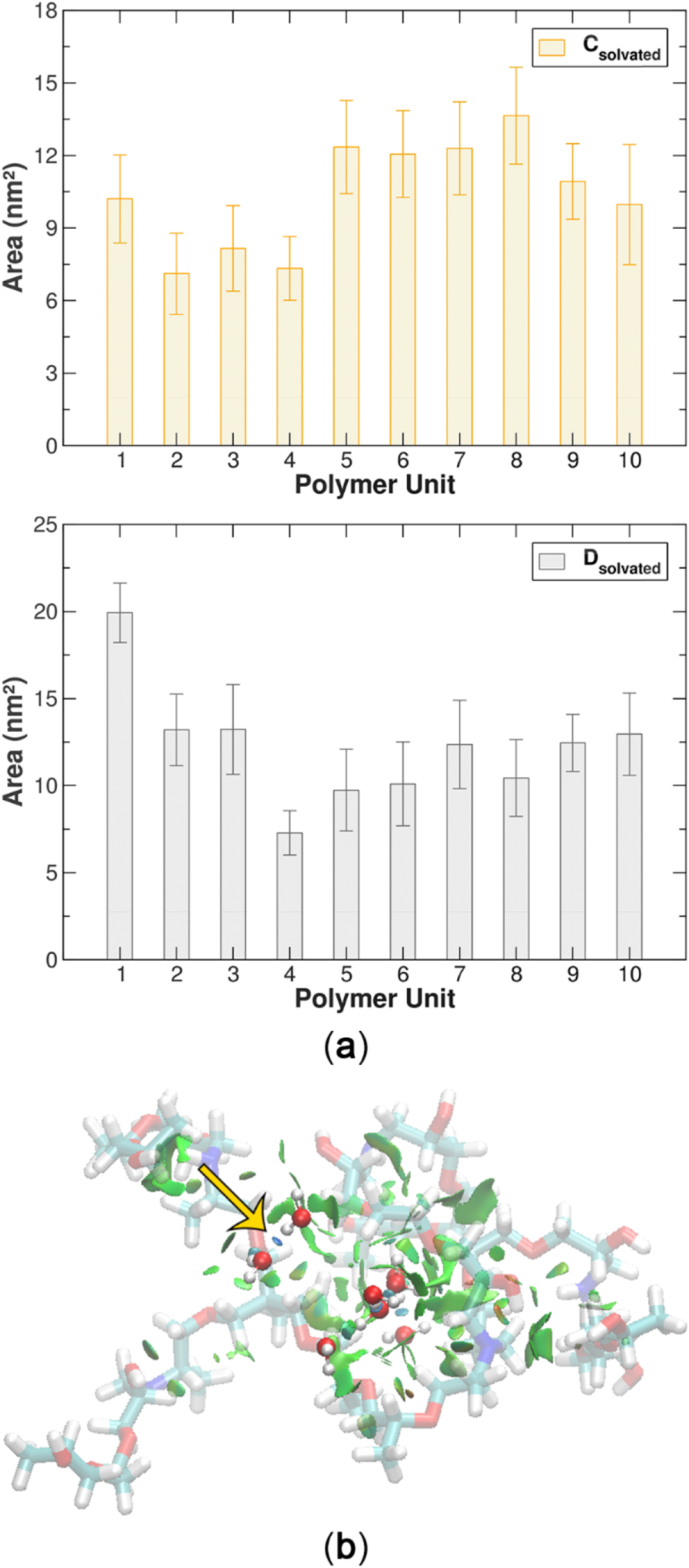
(a) Solvent accessible surface area (SASA) per MEP113 unit in **C**_**solvated**_ (single vesicle) and **D**_**solvated**_ (two vesicles) systems, showing differences in polymer exposure to solvent. (b) Noncovalent interaction (NCI) topology for a representative complex (MEP113 + 8 H_2_O) from **A**_**solvated**_, highlighting hydrogen bonds (blue surfaces) and van der Waals contacts (green surfaces) around the polymer structure.

To further characterize molecular interactions, RDF analyses of specific atomic pairs within MEP113 and between MEP113 and water were conducted (Figures S15–S19). The MEP113–MEP113 interactions are predominantly stabilized by three key hydrogen bonds: (i) MEP113–N­(central)····HO­(middle)–MEP113, (ii) MEP113–HO­(middle)····HN­(middle)–MEP113, and (iii) MEP113–HN­(middle)····HO­(middle)–MEP113. Additionally, MEP113–H_2_O interactions are governed by four primary hydrogen bonding patterns: (i) MEP113–OH­(terminal)····OH_2_, (ii) MEP113–HO­(terminal)····H–OH, (iii) MEP113–OH­(middle)····OH_2_, and (iv) MEP113–HO­(middle)····H–OH. These interactions play a crucial role in dictating molecular arrangement and stability in solution.

To complement the RDF findings, the noncovalent interaction (NCI) methodology^22^ was applied to analyze the interactions between MEP113 and water in a selected complex (MEP113 + 8 H_2_O) extracted from system **A**_**solvated**_ (Figure 4b). The NCI analysis highlights the presence of attractive interactions such as MEP113–(ether O)····H–OH and H–OH····OH_2_ (blue surfaces), along with weak van der Waals interactions: MEP113–CH····OH_2_ (green surfaces). These results reinforce the role of hydrogen bonding and van der Waals forces in stabilizing MEP113 in aqueous environments, further explaining the structural behavior observed in the simulations. Such interaction motifs directly underpin the macroscopic performance of these gels, including water uptake, porosity development, and the ability to carry micronutrients or bioactive molecules, strengthening the structure–function relationship proposed in this work.

Overall, the structural analysis illustrates that the formation of stable MEP113 assemblies is governed by a balance between intermolecular interactions and hydration effects. Dry systems promote molecular aggregation, whereas solvation facilitates dispersion through noncovalent interactions, which ultimately influence the stability and formation of the vesicle structures observed in aqueous environments.

### Energetic Analysis

We analyzed the average Coulomb and Lennard–Jones (LJ) interaction energies (kJ mol^–1^) between the components (polymer structure and water molecules) present in the systems **A**–**C** and **A**_**solvated**_–**D**_**solvated**_ during the final 0.5 ns (500 ps) or 25 ns (25000 ps) of NVT/NPT equilibration at 300 K. A summary of these results is provided in Figure S20. For systems without water molecules (**A**, **B**, and **C**), the obtained energy values were normalized by the number of polymer units. Conversely, for solvated systems (**A**_**solvated**_, **B**_**solvated**_, **C**_**solvated**_, and **D**_**solvated**_), the energy values were divided by the number of water molecules present in each system.

In dry conditions (systems **A**–**C**), we found that both Coulomb and Lennard–Jones interaction energies become progressively more negative per polymer unit as the number of MEP113 chains increases (Coulomb energy = −1106.670 → −1129.250 → −1157.690 kcal mol^–1^; LJ = −129.642 → −199.730 → −305.426 kcal mol^–1^). This trend indicates that increasing polymer concentration enhances interchain interactions, promoting aggregation and closer contact between chains. In system **A** (1 MEP113), interactions are limited to intrachain contributions, resulting in less negative energy values. In contrast, systems **B** and especially **C** display stronger and more favorable intermolecular interactions, as reflected by significantly more negative energies per unit. These observations highlight the role of polymer aggregation in stabilizing the system energetically.

Among the solvated structures, **C**_**solvated**_ demonstrates the most negative Coulomb and Lennard–Jones MEP113····MEP113 (Coulomb = −0.622 compared with −0.185 and −0.357 kcal mol^–1^; LJ = −0.189 regarding −0.032 and −0.073 kcal mol^–1^) and MEP113····Water energy values (Coulomb = −0.535 in relation to −0.199 and −0.394 kcal mol^–1^; LJ = −0.110 compared with −0.054 and −0.094 kcal mol^–1^), which can be attributed to its higher polymer concentration and an increased number of polymer–water contacts. This trend highlights the effective formation of hydration shells around the polymer chains, enhancing the stability and interaction profile of the solvated systems. The data underscore the crucial role of the solvent in modulating the interactions within these systems.

In the **D**_**solvated**_ system, which contains two independent polymer aggregates, the Coulomb interaction energies per water molecule for both MEP113····MEP113 (−0.626 kcal mol^–1^) and MEP113····Water (−0.542 kcal mol^–1^) are more negative than those observed in **C**_**solvated**_ (−0.622 and −0.535 kcal mol^–1^, respectively). This indicates that despite the spatial separation between the two aggregates, **D**_**solvated**_ favors electrostatic stabilization both within the polymer aggregates and between polymers and water molecules. Regarding Lennard–Jones interactions, **D**_**solvated**_ shows a more favorable (more negative) energy only for MEP113····Water (−0.120 kcal mol^–1^) interactions compared with **C**_**solvated**_ (−0.110 kcal mol^–1^), while the MEP113····MEP113 Lennard–Jones contribution remains slightly less favorable (**D**_**solvated**_ = −0.178 kcal mol^–1^; **C**_**solvated**_ = −0.189 kcal mol^–1^). These results suggest that the distribution of polymer aggregates in **D**_**solvated**_ does not compromise and, in some cases, enhances specific interaction types, particularly electrostatic interactions, while maintaining a stable hydration environment. The results show that in the **D**_**solvated**_ system, both the Coulomb and the Lennard–Jones interaction energies between polymers and between polymers and water molecules are much stronger (more negative) than in the less concentrated **B**_**solvated**_ system. This indicates that having a higher amount of polymer and a specific spatial arrangement of the aggregates improves the stability of the system by enhancing the interactions between its components.

Altogether, these energetic analyses corroborate the structural and dynamic observations from the 2D density maps, RDF profiles, and conformational parameters, reinforcing that solvation promotes the dispersion and stabilization of polymer chains through favorable electrostatic and van der Waals interactions, which are progressively modulated by the polymer concentration and organization within the system.

## Conclusions

We used molecular dynamics simulations to test the hypothesis that the self-assembly mechanism of MEP113 gel-forming units arises from specific hydration patterns and noncovalent interactions that cannot be resolved experimentally. This work represents the first molecular-scale characterization of the solvation-driven self-assembly process in amine–epoxide gels. Our simulations reveal previously undescribed structural regimes, including hydration-induced expansion, concentration-dependent packing transitions, and the stabilization of vesicle-like aggregates in explicit solvent. The findings confirm that polymer concentration and solvation govern the aggregation behavior of MEP113, with dry systems promoting polymer clustering and aqueous conditions facilitating molecular dispersion due to hydration effects, a mechanism directly relevant to understanding hydration-mediated organization in soft polymeric materials, nanogels, and responsive polymer networks. Radial distribution function analyses showed that water molecules disrupt polymer–polymer interactions while simultaneously organizing around polymer chains, especially at lower concentrations.

The investigation of structural parameters, including radius of gyration and end-to-end distance, revealed that MEP113 structures tend to adopt a more compact conformation compared with the system in hydrated conditions, driven by favorable water-mediated interactions. Hydrogen bonding and van der Waals contacts between the MEP113 units and water molecules played a pivotal role in stabilizing the resulting assemblies. Energetic analyses corroborated these findings, highlighting the role of electrostatic and dispersive interactions in modulating system stability according to the concentration and solvation state. These microscopic rearrangements directly explain macroscopic gel properties, such as swelling, porosity formation, and the capacity to encapsulate and release nutrients or bioactive molecules.

Overall, this study advances the understanding of the solvation-driven polymer self-assembly process under equilibrium conditions and provides an explicit structure–function framework that links molecular interactions to the emergent properties of amine–epoxide gels. This framework enables the rational design of polymer-based vesicles with tailored swelling, stability, and release profiles, directly benefiting applications in controlled nutrient delivery, as well as broader nanotechnological and biomedical engineering.

## Computational and Experimental Methodology

Molecular dynamics (MD) simulations were performed by following the workflow shown in Figure S1. MEP113 was described using the CHARMM36 force field,^23–27^ with parameters generated via the charmm2gmx tool.^28^ Water was modeled using TIP4P–2005,^29^ except for the **D**_**solvated**_ system, where SPC/E^30^ was employed to reduce computational cost. Both models adequately reproduce key structural and dynamical properties of liquid water.^31–33^ Initial configurations were constructed with Packmol.^34^ Simulation boxes measured 8 × 8 × 8 nm^3^ (**A**–**C**, **A**_**solvated**_, **B**_**solvated**_), 15 × 15 × 15 nm^3^ (**C**_**solvated**_), and 20 × 20 × 60 nm^3^ (**D**_**solvated**_). All simulations were carried out using GROMACS 2024.4.^35^

Energy minimization was performed using the steepest descent, followed by the conjugate gradient method. Systems were equilibrated under NVT conditions (100 ps at 750 K and 100 ps at 300 K), followed by further minimization and a 500 ps NVT equilibration at 300 K. Subsequent NPT equilibrations at 1.0 bar were system dependent: 25 ns for **B** and **C**; two 25 ns steps for **A**_**solvated**_ and **B**_**solvated**_; and 100 ns for **C**_**solvated**_ and **D**_**solvated**_. Hydrogen bonds were constrained using the LINCS algorithm.^36^

Temperature coupling employed the Nosé–Hoover thermostat^37,38^ or the v–rescale thermostat,^39^ while pressure control was achieved using the Parrinello–Rahman^40,41^ or c-rescale^42^ barostats, as more detailed in Figure S2. Long-range electrostatics were treated with the PME method^43^ using cutoffs of 1.2 or 1.4 nm, and short-range interactions were handled via the Verlet cutoff scheme.^44^

Structural and energetic analyses (Figure S3) included radial distribution functions, radius of gyration, end-to-end distance, vesicle diameter, 2D density maps, and Coulomb and Lennard–Jones interaction energies, all computed with GROMACS tools.^35^ Visualization was performed using VMD,^45^ and noncovalent interactions between MEP113 and water molecules were analyzed using the NCI method.^22^

Amine–epoxide colloidal particles were synthesized via a two-step procedure adapted from the literature.^17,46^ Initially, trimethylolpropane tris­[poly­(propylene glycol), amine-terminated] ether and diepoxy poly­(ethylene glycol) (Mw ≈ 500 g mol^–1^) were dissolved in water (15 wt %) and incubated at 75 °C for 15 min to form a water-soluble prepolymer. The solution was then diluted to 0.5 wt % and incubated for 30 min to induce colloid formation.^17,46^ Particle morphology was characterized by transmission electron microscopy (TEM) using a JEOL JEM-1400 Flash instrument operating at 100 kV.

## Supplementary Material











## Data Availability

The Repository of simulation data is available from https://github.com/molmodcs/Polyetheramine-Epoxide-Gels-Molecular-Dynamics.git.

## Notes

All details regarding the computational methodology applied in the present molecular simulations; details on the preparation and TEM characterization of the gels; potential energy per molecule throughout geometry optimization using the CG method; (iv) kinetic, potential, and total energy per molecule, and density (only solvated systems) along with the last NVT/NPT equilibration; radial distribution functions (RDFs) of specific atomic pairs regarding polymer–polymer, polymer–water, and water–water interactions for all investigated systems; average and standard deviation values associated with *R*_g_ and end-to-end distance (PDF)
Structures (GRO files) related to the last NVT/NPT equilibration, for all systems investigated (ZIP)
MDPs files (ZIP)
TOP and ITP files (ZIP)
Videos (GIF files) related to the last NVT/NPT equilibration, for all systems investigated (ZIP)
