# Supplementary material for: Solvation-Driven Self-Assembly of Polyetheramine–Epoxide Gels: Insights from Molecular Simulations
Source: ACS Polym Au. 2025 Dec 30;6(1):405–14. doi: 10.1021/acspolymersau.5c00159 (PMC12903512; doi:10.1021/acspolymersau.5c00159)
Supplement: Supplementary file 1 [file lg5c00159_si_001.pdf]

Solvation–Driven Self–Assembly of Polyetheramine–Epoxide Gels: Insights from Molecular Simulations

Renato P. Orenha,[1*] Eduardo F. Molina,[1*] Felipe B. Alves,[1] Bruno A. Fico,[1] Marcelo Albuquerque[2,3] Renato L. T. Parreira,[1] and Luciano T. Costa[3*]

[1] Núcleo de Pesquisas em Ciências Exatas e Tecnológicas, Universidade de Franca, 14404-600, Franca, SP, Brazil. Email: rorenha@unifran.edu.br / eduardo.molina@unifran.edu.br

[2] Institute of Physics, Universidade Federal Fluminense, Av. Gal. Milton Tavares de Souza, 24210-346, Niterói, RJ, Brazil.

[3] Departamento de Química, Universidade Federal Fluminense, 24020-150, Outeiro de São João Batista, RJ, Brazil. Email: ltcosta@id.uff.br

Supplementary Material

Summary

	Page
**Computational Methods**	S3
**Figure S1.** Workflow of the computational protocol used: force field parametrization, system construction, energy minimization, and equilibration (NVT and NPT steps).	S4
**Figure S2.** Computational workflow and parameters used in the simulations, including force field (CHARMM36), water models (TIP4P–2005, SPC/E), cutoff schemes, and thermostats/barostats.	S5
**Figure S3.** Overview of structural and energetic parameters analyzed: i) radial distribution functions (RDFs), ii) radius of gyration (Rg), iii) end–to–end distance, iv) vesicle diameter, v) 2D density maps, vi) Coulomb and Lennard–Jones interaction energies, and vii) Non–Covalent Interaction (NCI) analysis between MEP113 and water molecules. These analyses together allowed us to correlate polymer organization, solvation effects, and vesicle stability at the molecular level.	S6
**Experimental Methodology**	S6
**Figure S4.** Potential energy per molecule (kJ mol^{-1}) during geometry optimization using the conjugate gradient (CG) method for isolated polymer aggregates (**A**–**C**) and for solvated systems ($\mathbf{A_{solvated}}$–$\mathbf{D_{solvated}}$), confirming energy minimization and structural stability before equilibration.	S7
**Figure S5.** Kinetic, potential, and total energy per molecule (kJ mol^{-1}) for system **A** after its respective equilibration steps at 300 K. Stability is evidenced by convergence of the energy values over time.	S8
**Figure S6.** Kinetic, potential, and total energy per molecule (kJ mol^{-1}) for system **B** after its respective equilibration steps at 300 K. Stability is evidenced by convergence of the energy values over time.	S8
**Figure S7.** Kinetic, potential, and total energy per molecule (kJ mol^{-1}) for system **C** after its respective equilibration steps at 300 K. Stability is evidenced by convergence of the energy values over time.	S9
**Figure S8.** Kinetic, potential, and total energy per molecule (kJ mol^{-1}), together with system density (kg m^{-3}), for solvated system $\mathbf{A_{solvated}}$. The data confirm equilibration and density stabilization under NPT conditions at 300 K.	S9

**Figure S9.** Kinetic, potential, and total energy per molecule (kJ mol^{-1}), together with system density (kg m^{-3}), for solvated system $\mathbf{B_{solvated}}$. The data confirm equilibration and density stabilization under NPT conditions at 300 K.	S10
**Figure S10.** Kinetic, potential, and total energy per molecule (kJ mol^{-1}), together with system density (kg m^{-3}), for solvated system $\mathbf{C_{solvated}}$. The data confirm equilibration and density stabilization under NPT conditions at 300 K.	S10
**Figure S11.** Kinetic, potential, and total energy per molecule (kJ mol^{-1}), together with system density (kg m^{-3}), for solvated system $\mathbf{D_{solvated}}$. The data confirm equilibration and density stabilization under NPT conditions at 300 K.	S11
**Figure S12.** Radial distribution functions (RDFs) for $\mathbf{A_{solvated}}$–$\mathbf{D_{solvated}}$ systems: i) polymer–water interactions (left) and ii) water–water interactions (right). The profiles show the formation of hydration shells around the polymer and the preservation of bulk water hydrogen–bond network.	S12
**Figure S13.** Histograms of the distribution of radius of gyration (Rg) of MEP113 structures in systems **A**–**C** and $\mathbf{A_{solvated}}$–$\mathbf{D_{solvated}}$. These data quantify conformational flexibility depending on solvation.	S13
**Figure S14.** Histograms of the distribution of end–to–end distance of MEP113 structures in systems **A**–**C** and $\mathbf{A_{solvated}}$–$\mathbf{D_{solvated}}$. These data quantify compaction/extension trends depending on solvation.	S14
**Figure S15.** Radial distribution functions (RDFs) between specific atoms of MEP113 units in dry systems (**A**–**C**). These results reveal preferred intramolecular and intermolecular contacts under different conditions.	S15
**Figure S16.** Radial distribution functions (RDFs) between specific atoms of MEP113 units in dry systems (**A**–**C**). These results reveal preferred intramolecular and intermolecular contacts under different conditions.	S16
**Figure S17.** Radial distribution functions (RDFs) between specific atoms of MEP113 units in solvated systems ($\mathbf{A_{solvated}}$–$\mathbf{D_{solvated}}$). These results reveal preferred intramolecular and intermolecular contacts under different conditions.	S17
**Figure S18.** Radial distribution functions (RDFs) of polymer–water atomic pairs in solvated systems, highlighting hydrogen–bonding interactions between hydroxyl/amine groups of MEP113 and surrounding water molecules, key to solvation and stability.	S18
**Figure S19.** Radial distribution functions (RDFs) of polymer–water atomic pairs in solvated systems, highlighting hydrogen–bonding interactions between hydroxyl/amine groups of MEP113 and surrounding water molecules, key to solvation and stability.	S19
**Figure S20.** Average Coulomb and Lennard–Jones interaction energies (kJ mol^{-1}) between polymer units and between polymer–water pairs in systems **A**–**C** and $\mathbf{A_{solvated}}$–$\mathbf{D_{solvated}}$, calculated over the last equilibration windows. Normalization was done per polymer unit (dry systems) or per water molecule (solvated systems). The results highlight how solvation modulates electrostatic and dispersive stabilization.	S20
**Table S1.** Average and standard deviation values of radius of gyration (Rg) and end–to–end distance (nm) for MEP113 in systems **A**–$\mathbf{D_{solvated}}$. These parameters quantify molecular flexibility and complement the structural analyses and the NCI–based insights into polymer–water stabilization.	S21
**Repository of Simulation Data**	S22

Computational Methods

The protocol applied in the present investigation, presented in the Figure S1, starts with the use of the CHARMM36 force field parameters to describe MEP113.[23–27] The force field parameters for MEP113 were generated using the charmm2gmx software,[28] and water was modeled using the TIP4P–2005 model.[29] For the $\mathbf{D_{solvated}}$ system, we used the SPC/E water model[30] to reduce computational cost because the relevantly larger number of atoms regarding the other systems investigated in the present study. Overall, TIP4P–2005 and SPC/E models mimic, to some extent, key structural and dynamical properties of water in the condensed phase, including the hydrogen–bond network, radial and angular distribution functions, density, and diffusion coefficient.[31] The choice between the two water models is dependent on the specific research question and the desired balance between accuracy and computational efficiency.[32,33] All systems were built using the Packmol software, which employs an objective function to optimize the distances between particles in a box, keeping them within an acceptable tolerance range.[34] The box sizes for the systems investigated were: **A**–**C**, $\mathbf{A_{solvated}}$ and $\mathbf{B_{solvated}}$) 8 x 8 x 8; $\mathbf{C_{solvated}}$) 15 x 15 x 15; and $\mathbf{D_{solvated}}$) 20 x 20 x 60 nm^3. Molecular dynamics (MD) simulations were conducted using the GROMACS 2024.4 package.[35]

Following with the calculation's routine, see Figure S1, we performed energy minimization using the steepest descent method followed by the conjugate gradient method. We carried out two NVT equilibration steps. The first one for 100 ps at 750 K, and the second for 100 ps at 300 K. The resulting geometries were again subjected to energy minimization using the steepest descent method followed by the conjugate gradient method. For all systems, a 500 ps NVT equilibration at 300 K was performed. For systems **B** and **C** a 25 ns NPT equilibration at 1.0 bar was conducted. For systems $\mathbf{A_{solvated}}$ and $\mathbf{B_{solvated}}$ two 25 ns NPT equilibration at 1.0 bar were conducted. For systems $\mathbf{C_{solvated}}$ and $\mathbf{D_{solvated}}$, a 100 ns NPT equilibration at 1.0 bar was performed. We used the LINCS algorithm[36] to constrain the hydrogen bonds in systems **A**, $\mathbf{A_{solvated}}$ and $\mathbf{B_{solvated}}$ (only in the first NPT equilibration), and $\mathbf{C_{solvated}}$ and $\mathbf{D_{solvated}}$.

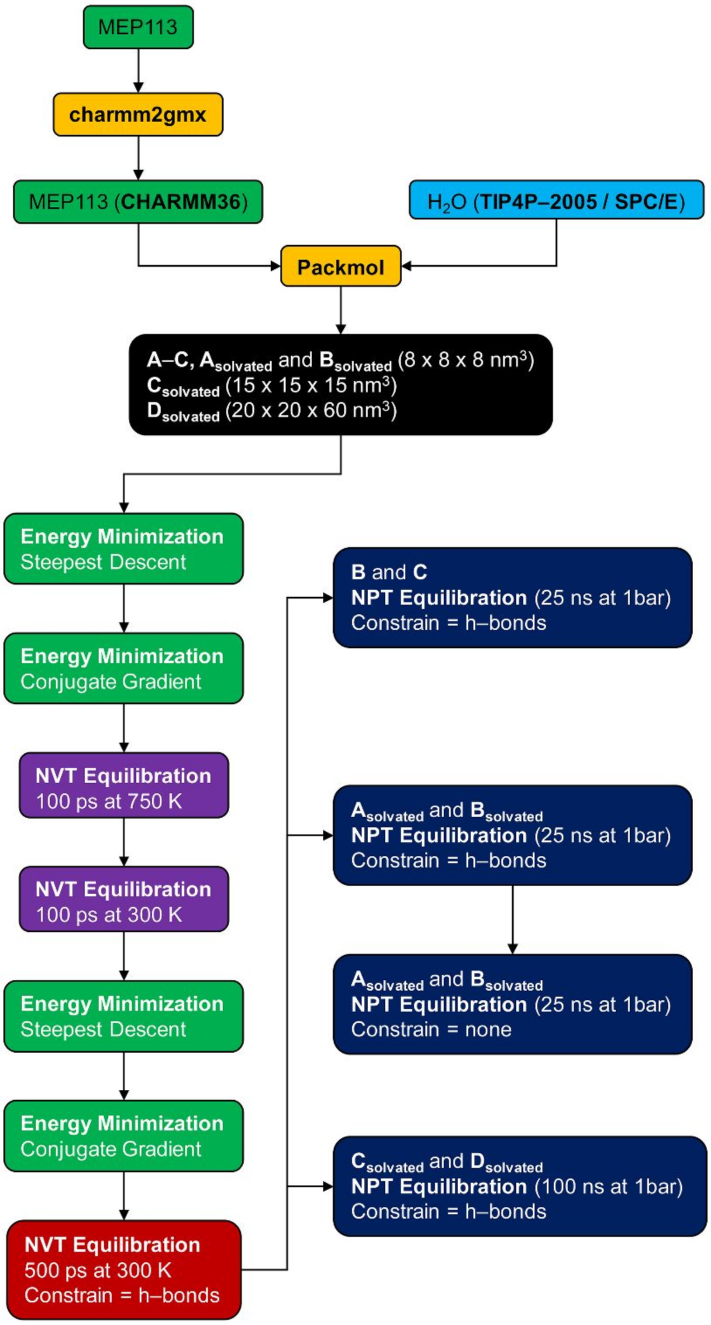

Figure S1. Workflow of the computational protocol used: force field parametrization, system construction, energy minimization, and equilibration (NVT and NPT steps).

Several details related to the parameters used in the simulations were organized in the Figure S2. This includes, for example, the temperature control in systems **B**, **C**, $\mathbf{A}_{\mathbf{solvated}}$, and $\mathbf{B}_{\mathbf{solvated}}$, which was performed using the Nose–Hoover[37,38] method with a coupling constant of 0.1 ps. For systems $\mathbf{C}_{\mathbf{solvated}}$ and $\mathbf{D}_{\mathbf{solvated}}$, the temperature was controlled by the v–rescale method[39] with a coupling constant of 1.0 ps. The pressure was controlled for systems $\mathbf{A}_{\mathbf{solvated}}$–$\mathbf{C}_{\mathbf{solvated}}$ a using the Parrinello–Rahman method[40,41] with a coupling constant of 5.0 ps. To systems **B**, **C** and $\mathbf{D}_{\mathbf{solvated}}$, the pressure was controlled through of the c–rescale method[42] coupling constant of 5.0

ps. Long–range electrostatic interactions were handled using the Particle Mesh Ewald (PME) method[43] with a cut–off of 1.2 (for systems **A**–**C**, **A**$_{\mathbf{solvated}}$, **B**$_{\mathbf{solvated}}$ and **D**$_{\mathbf{solvated}}$) or 1.4 (for system **C**$_{\mathbf{solvated}}$) nm. Short–range non–bonded interactions were treated with the Verlet cutoff–scheme[44] and a pair list radius of 1.2 (exceptionally 1.4 to **C**$_{\mathbf{solvated}}$) nm. Short–range van der Waals (VDW) interactions in real space were switched off at 1.2 (exceptionally 1.4 to **C**$_{\mathbf{solvated}}$) nm.

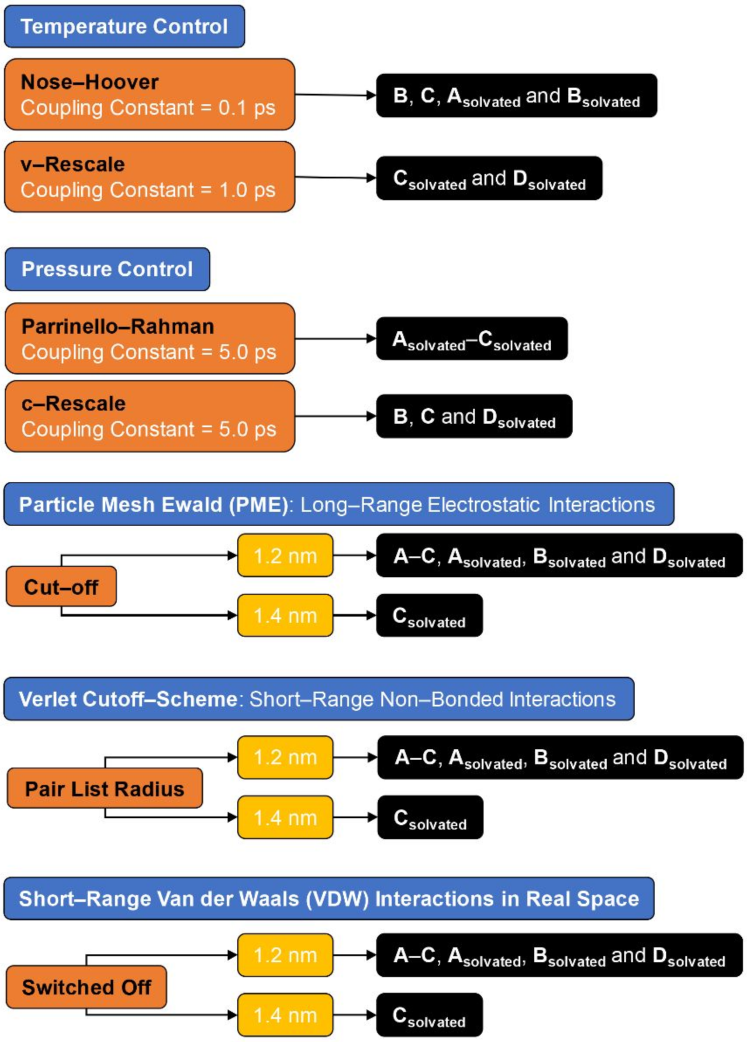

Figure S2. Computational workflow and parameters used in the simulations, including force field (CHARMM36), water models (TIP4P–2005, SPC/E), cutoff schemes, and thermostats/barostats.

The parameters used to analyze the systems in the present study are ordered in the Figure S3. Since that the structural properties were characterized using: i) radial distribution function; ii) radius of gyration; iii) head–to–head distance; and iv) average diameter of the vesicle. The density map of the vesicles (elaborated from MEP113 structures) also was evaluated. The energetic analysis was carried out by studying the average Coulomb and Lennard–Jones energies. All these analyses were performed using the GROMACS suite of tools.[35] The VMD molecular visualization software package was used for graphical visualization of the systems investigated.[45] The main non–covalent interactions present between the MEP113 structure and eight water

molecules (selected from systema $\mathbf{A}_{\mathbf{solvated}}$) were identified through of the NCI index visualization tool.[22]

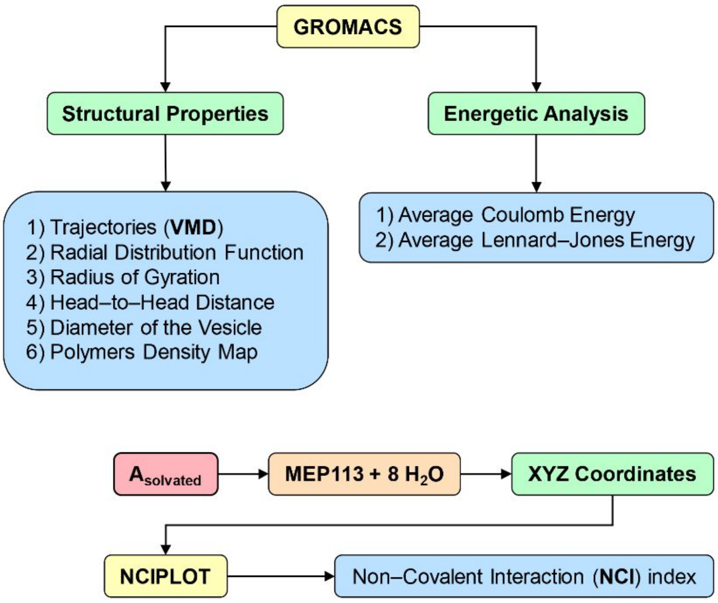

Figure S3. Overview of structural and energetic parameters analyzed: i) radial distribution functions (RDFs), ii) radius of gyration (Rg), iii) end–to–end distance, iv) vesicle diameter, v) 2D density maps, vi) Coulomb and Lennard–Jones interaction energies, and vii) Non–Covalent Interaction (NCI) analysis between MEP113 and water molecules. These analyses together allowed us to correlate polymer organization, solvation effects, and vesicle stability at the molecular level.

Experimental Methodology

Amine–epoxide colloid synthesis: Colloidal amine–epoxide particles were synthesized using a two–step procedure adapted from the literature.[17,46] In the first step, a trimethylolpropane tris[poly(propylene glycol), amine terminated] ether and Diepoxy Poly(ethylene glycol) (DPEG, $C_3H_5O_2$–$(C_2H_4O)_n$–C_3H_5O, average Mw = 500 g mol^{-1}) were dissolved in deionized water to obtain a total monomer concentration of 15 wt%. The solution was incubated under quiescent conditions in an isothermal water bath at 75 °C for 15 min, allowing the epoxy–amine reaction to proceed and yielding a water–soluble prepolymer intermediate. In the second step, the prepolymer solution was diluted with deionized water to a final concentration of 0.5 wt% and further incubated in the water bath for 30 min without agitation to promote colloid formation.[17,46]

Characterization: The morphologies of the colloidal particles were evaluated by transmission electron microscopy (TEM), using a JEM–1400 Flash instrument (JEOL) operating at 100 kV. For acquisition of the images, a small droplet of the aqueous formulation was deposited onto a carbon–coated copper grid.

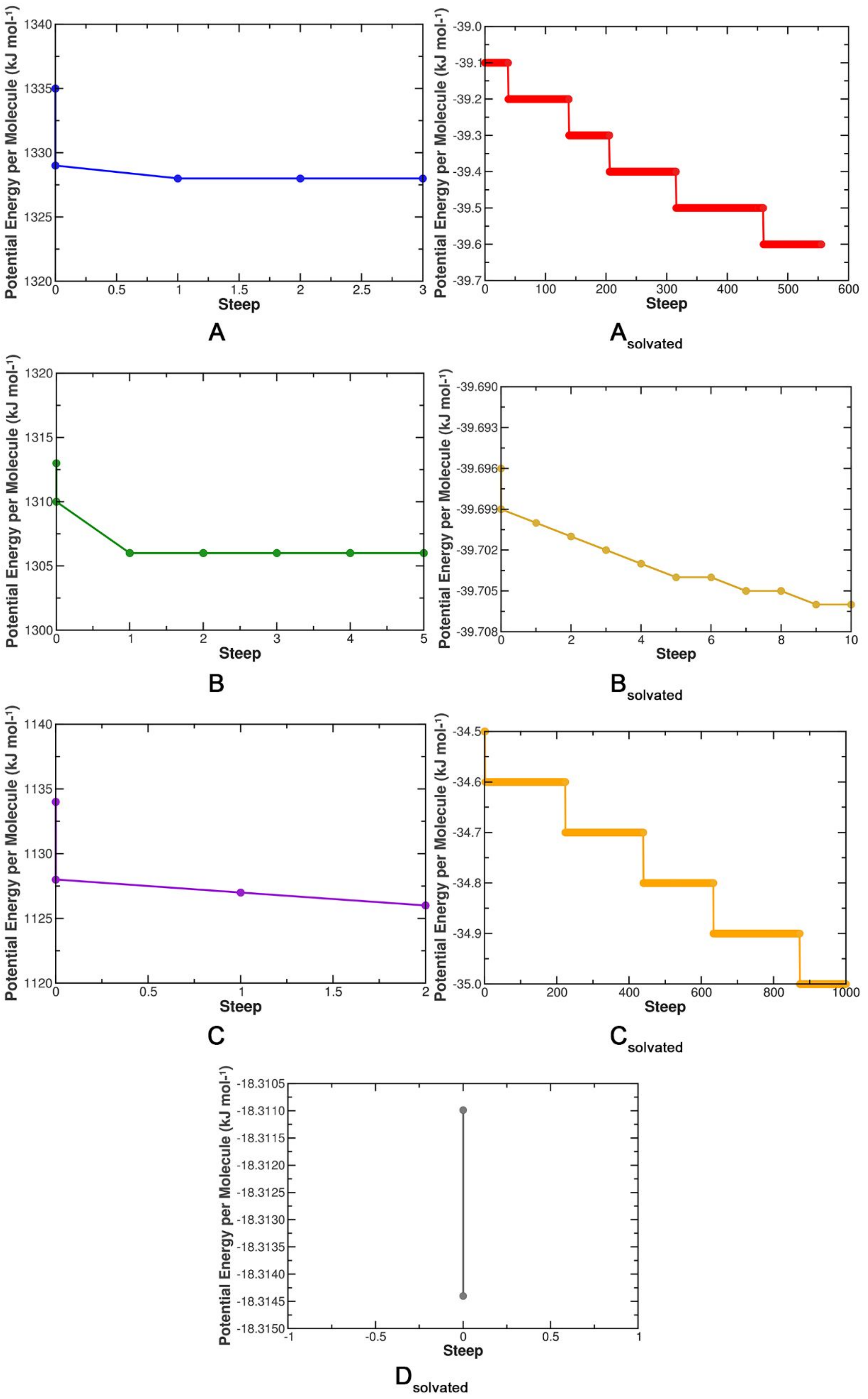

Figure S4. Potential energy per molecule (kJ mol^{-1}) during geometry optimization using the conjugate gradient (CG) method for isolated polymer aggregates (**A**–**C**) and for solvated systems (**A$_{solvated}$**–**D$_{solvated}$**), confirming energy minimization and structural stability before equilibration.

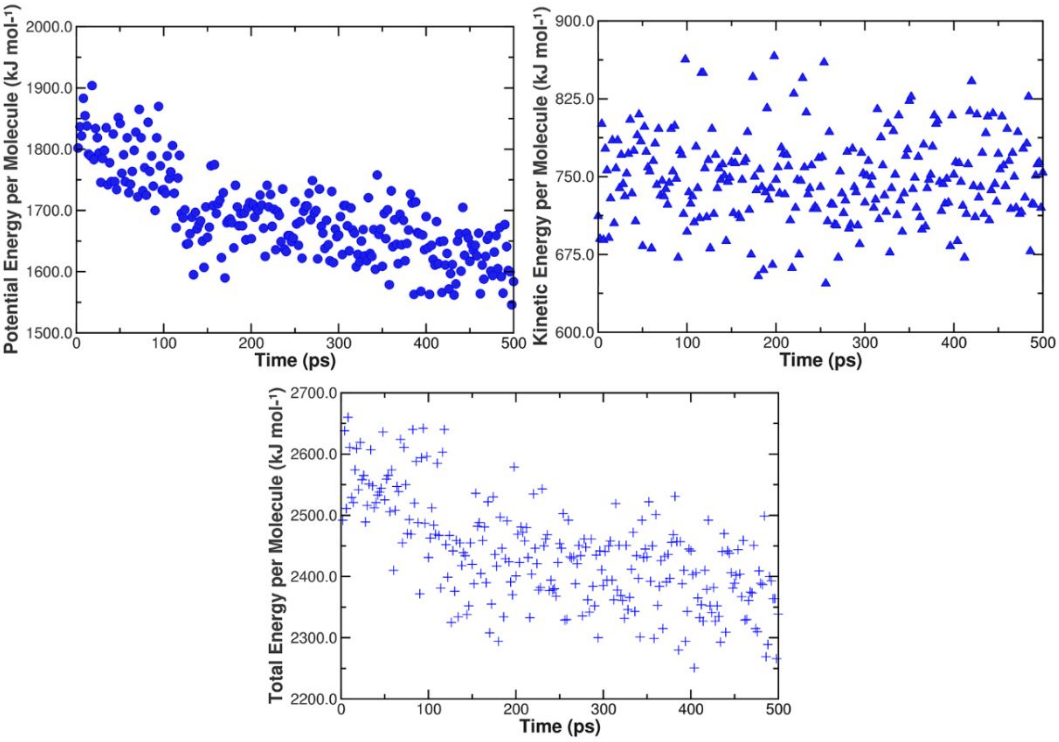

Figure S5. Kinetic, potential, and total energy per molecule (kJ mol^{-1}) for system **A** after its respective equilibration steps at 300 K. Stability is evidenced by convergence of the energy values over time.

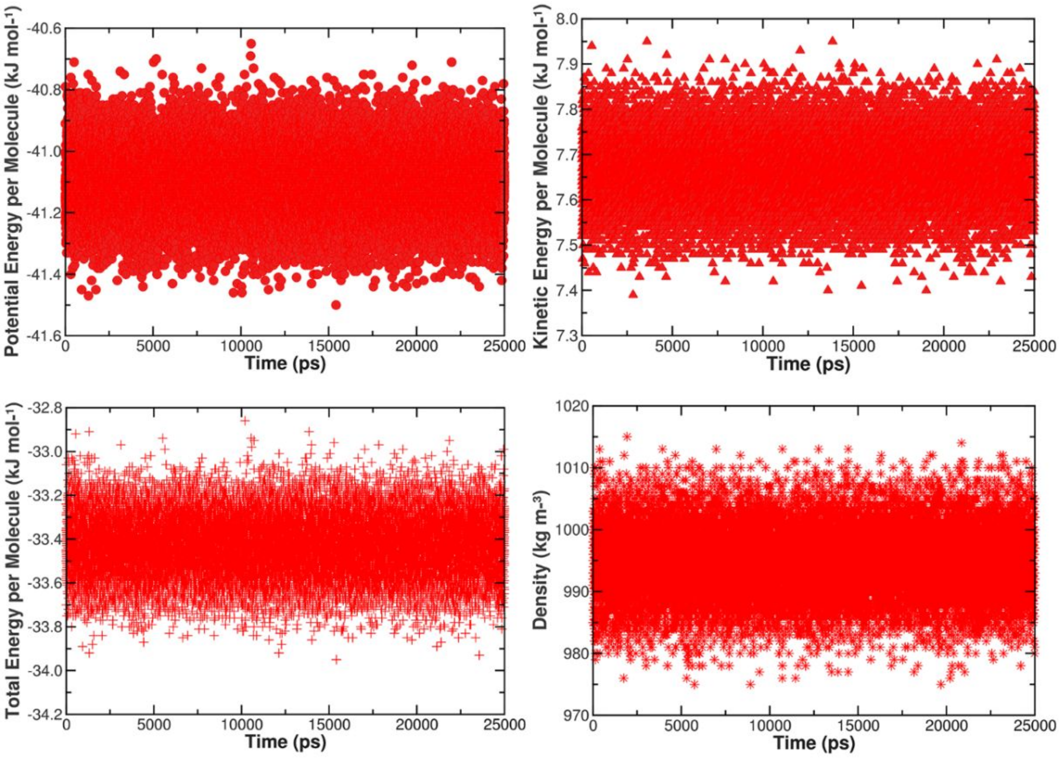

Figure S6. Kinetic, potential, and total energy per molecule (kJ mol^{-1}) for system **B** after its respective equilibration steps at 300 K. Stability is evidenced by convergence of the energy values over time.

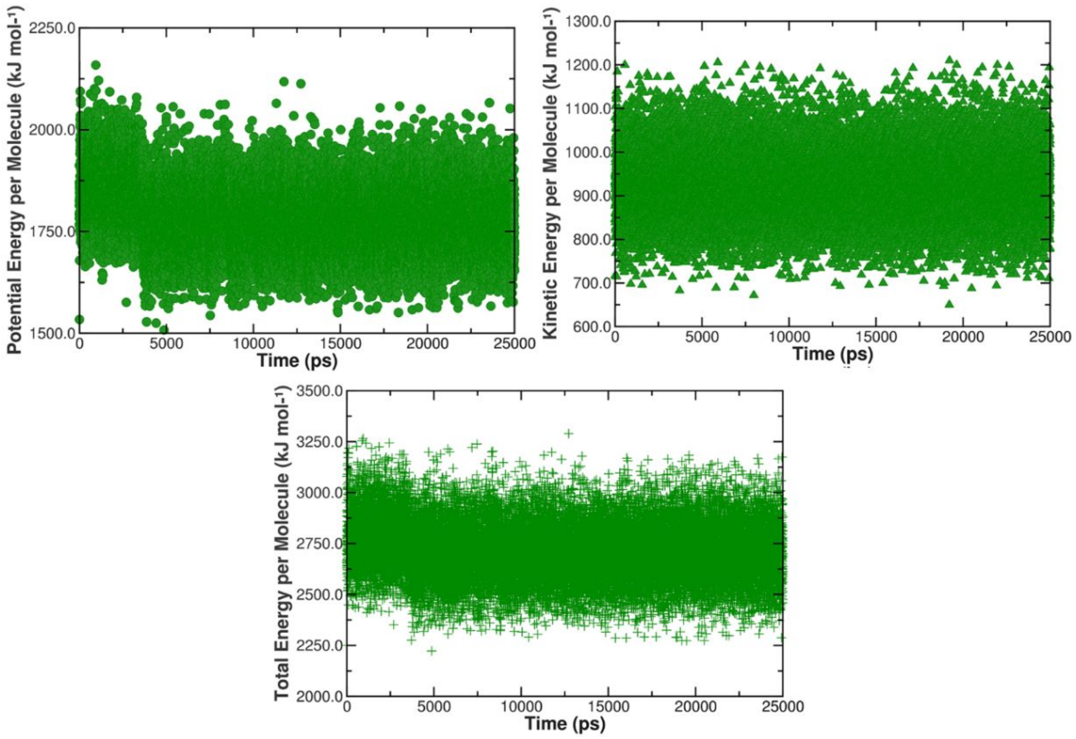

Figure S7. Kinetic, potential, and total energy per molecule (kJ mol^{-1}) for system **C** after its respective equilibration steps at 300 K. Stability is evidenced by convergence of the energy values over time.

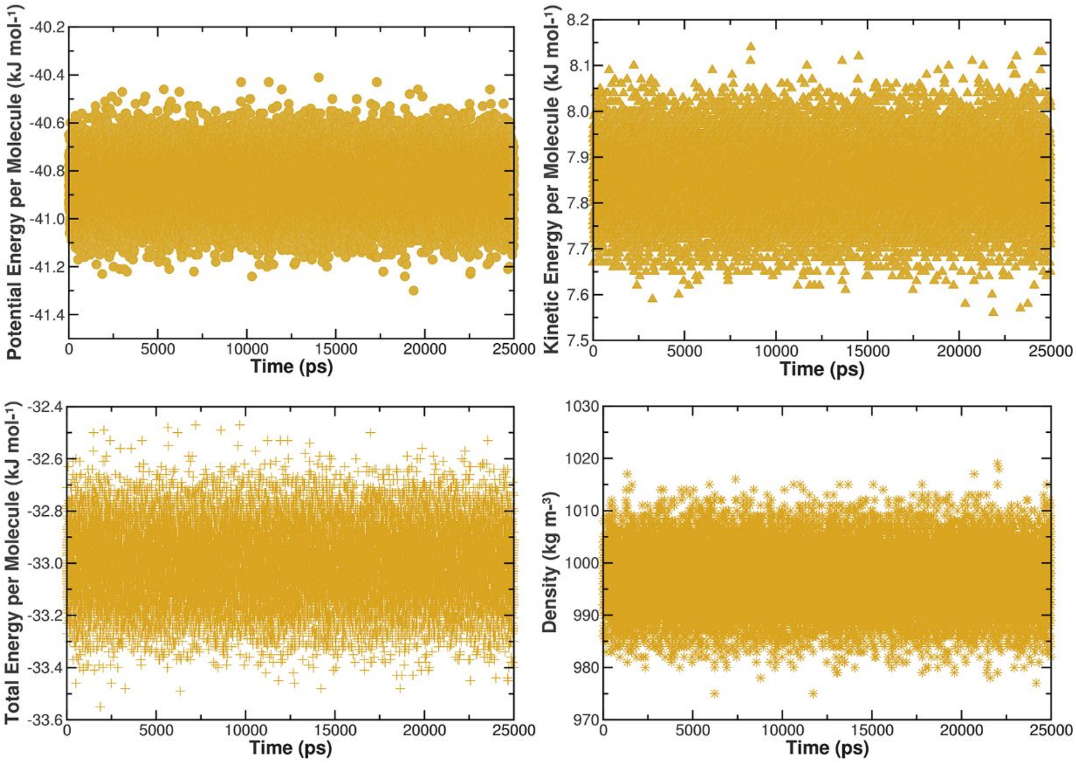

Figure S8. Kinetic, potential, and total energy per molecule (kJ mol^{-1}), together with system density (kg m^{-3}), for solvated system $\mathbf{A}_{\mathbf{solvated}}$. The data confirm equilibration and density stabilization under NPT conditions at 300 K

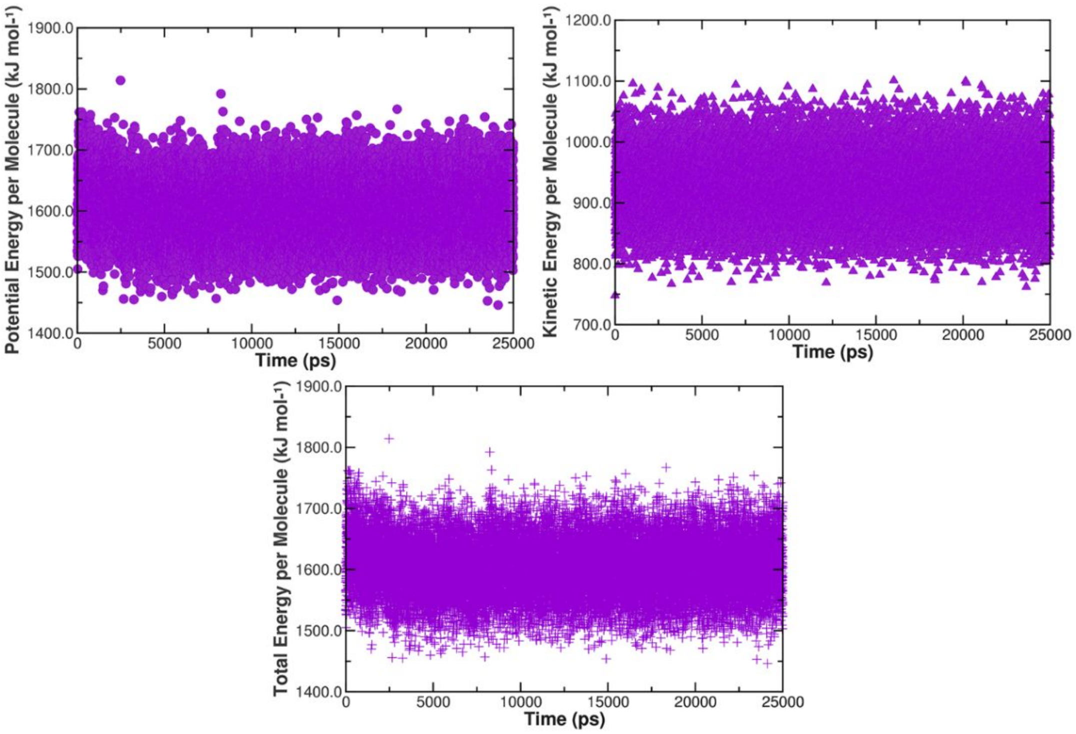

Figure S9. Kinetic, potential, and total energy per molecule (kJ mol^{-1}), together with system density (kg m^{-3}), for solvated system $\mathbf{B_{solvated}}$. The data confirm equilibration and density stabilization under NPT conditions at 300 K.

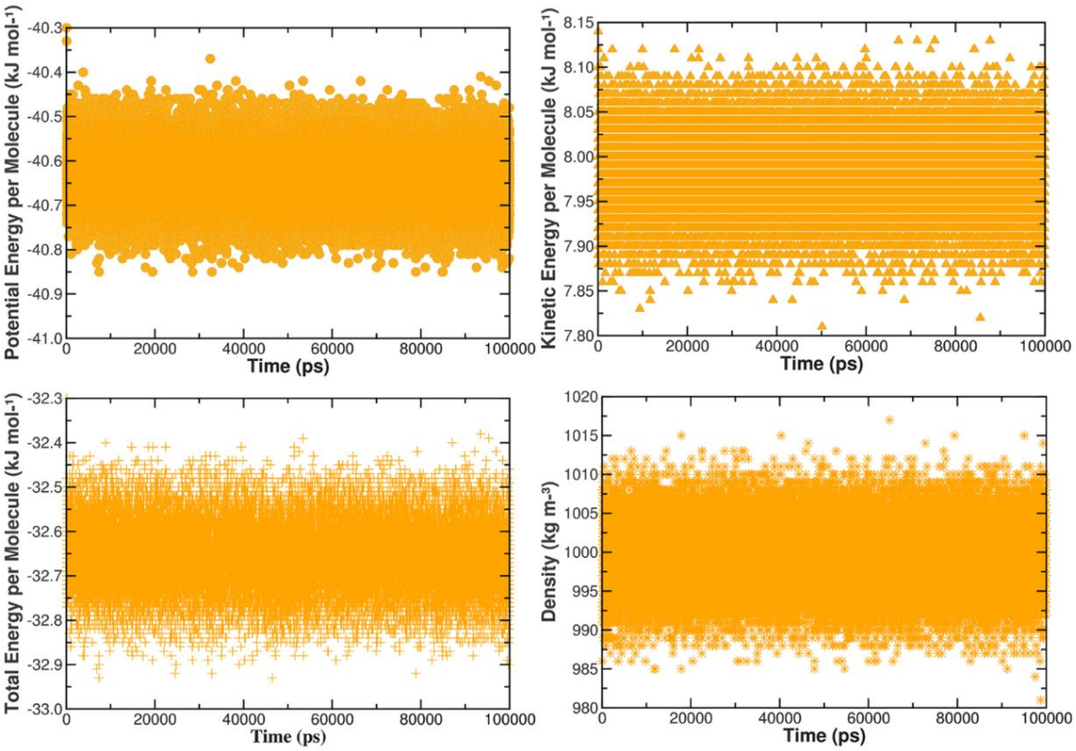

Figure S10. Kinetic, potential, and total energy per molecule (kJ mol^{-1}), together with system density (kg m^{-3}), for solvated system $\mathbf{C_{solvated}}$. The data confirm equilibration and density stabilization under NPT conditions at 300 K.

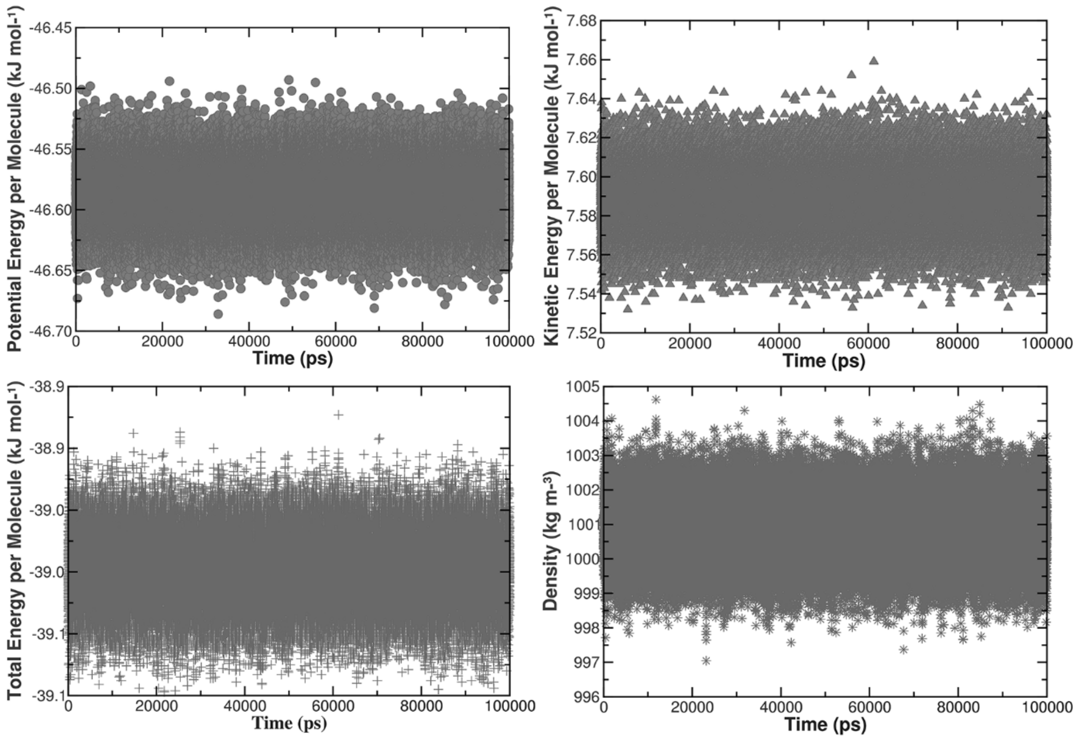

Figure S11. Kinetic, potential, and total energy per molecule (kJ mol^{-1}), together with system density (kg m^{-3}), for solvated system $\mathbf{D_{solvated}}$. The data confirm equilibration and density stabilization under NPT conditions at 300 K.

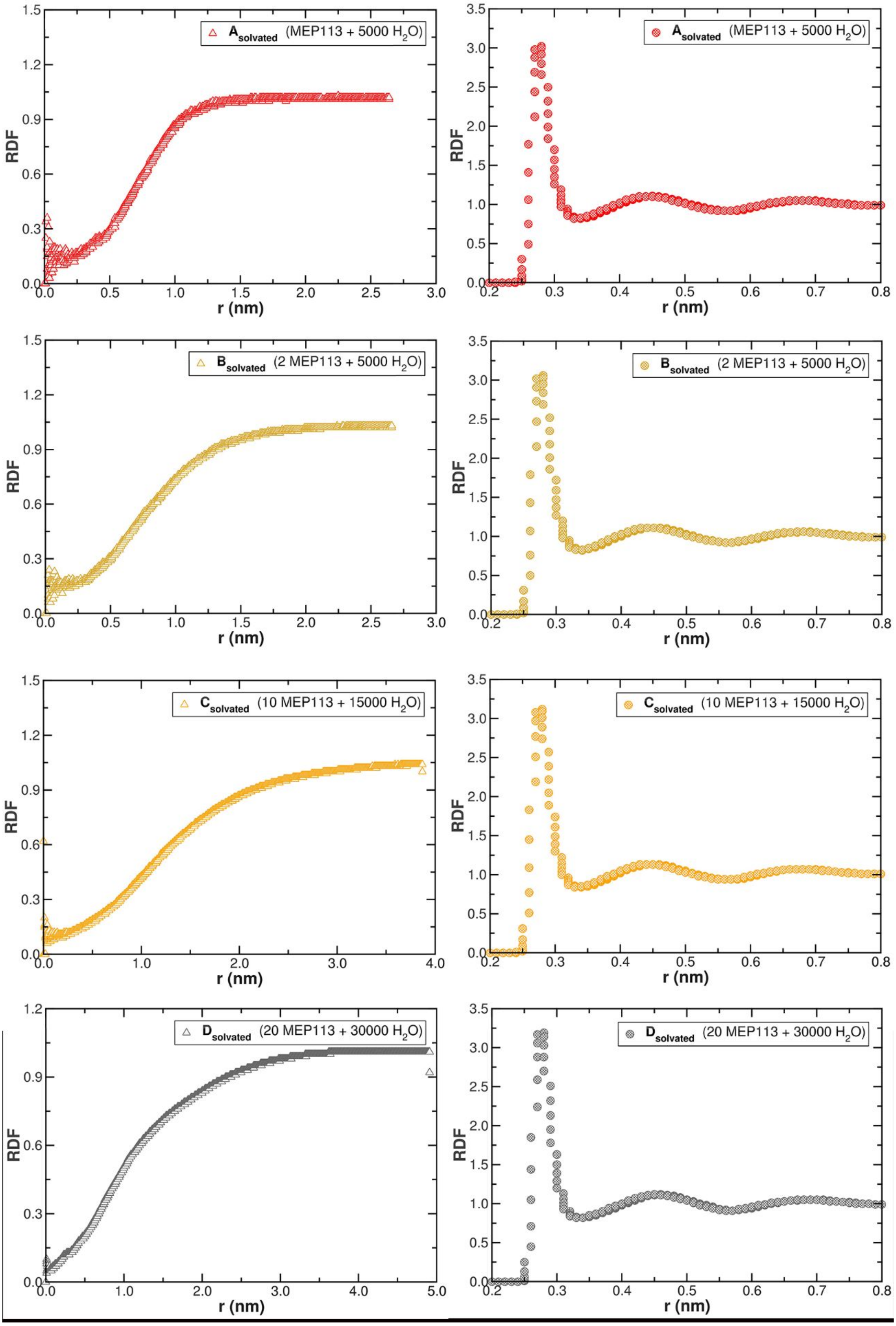

Figure S12. Radial distribution functions (RDFs) for **A$_{solvated}$**–**D$_{solvated}$** systems: i) polymer–water interactions (left) and ii) water–water interactions (right). The profiles show the formation of hydration shells around the polymer and the preservation of bulk water hydrogen–bond network.

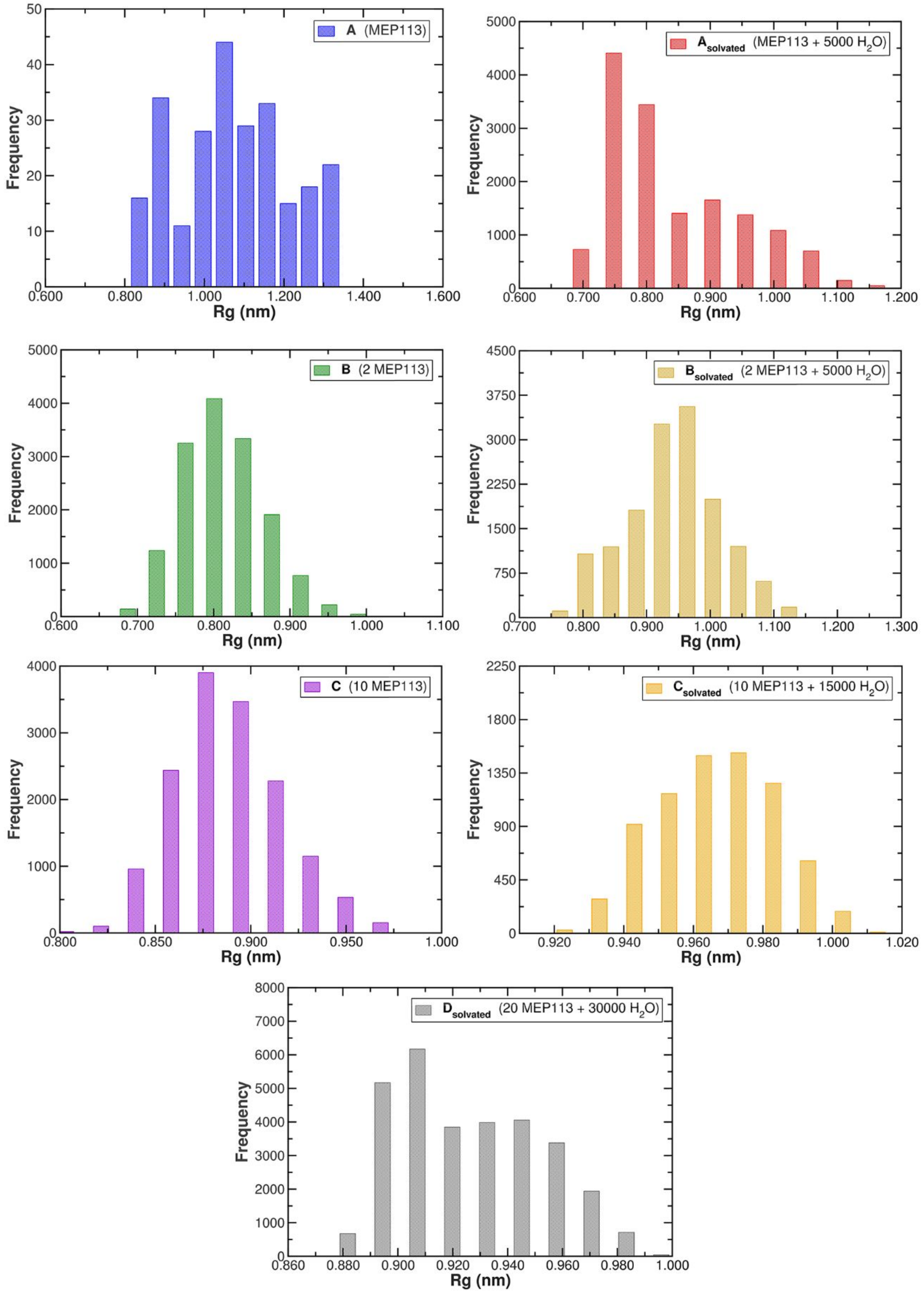

Figure S13. Histograms of the distribution of radius of gyration (Rg) of MEP113 structures in systems **A**–**C** and **A$_{solvated}$**–**D$_{solvated}$**. These data quantify conformational flexibility depending on solvation.

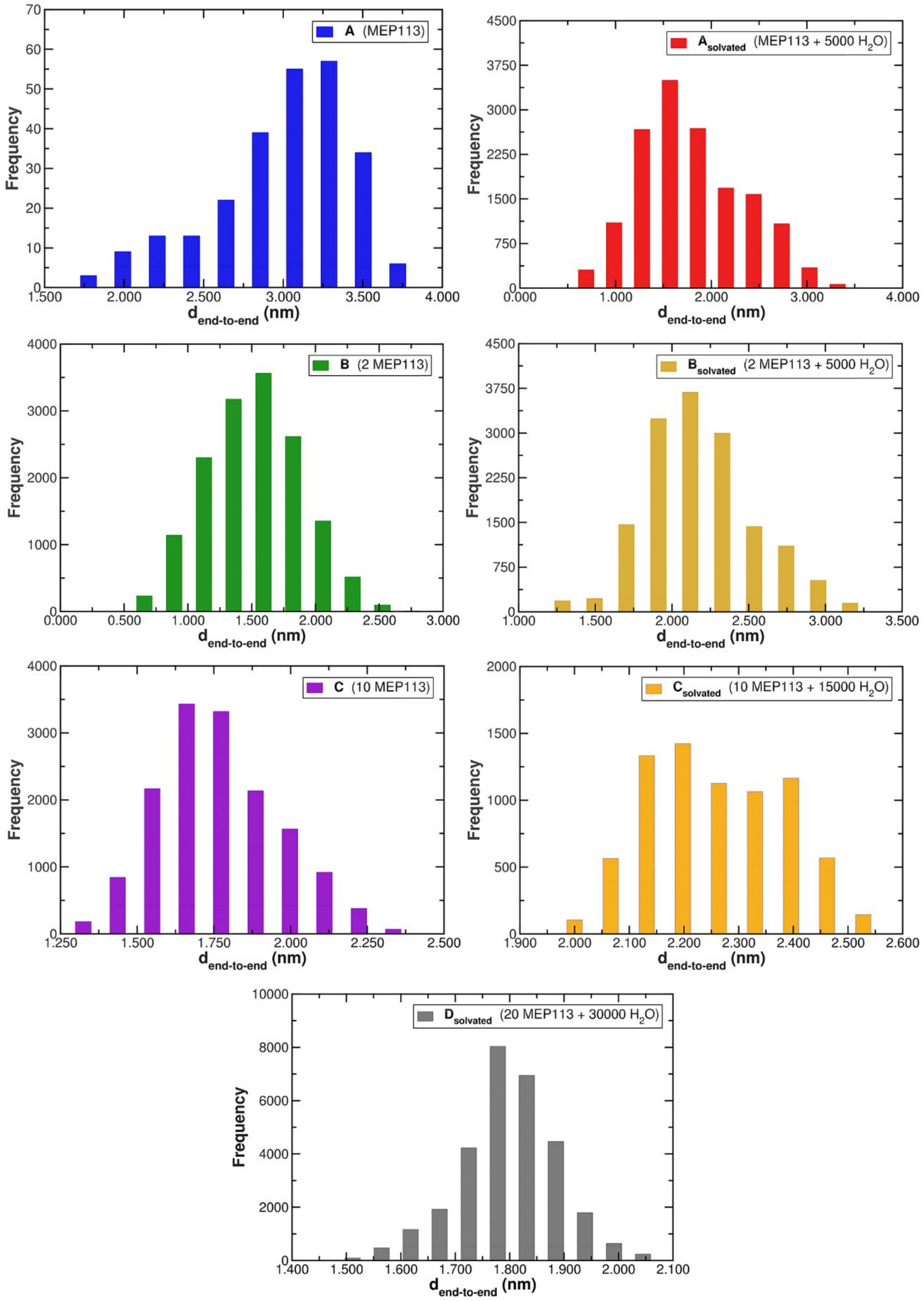

Figure S14. Histograms of the distribution of end–to–end distance of MEP113 structures in systems **A**–**C** and $\mathbf{A_{solvated}}$–$\mathbf{D_{solvated}}$. These data quantify compaction/extension trends depending on solvation.

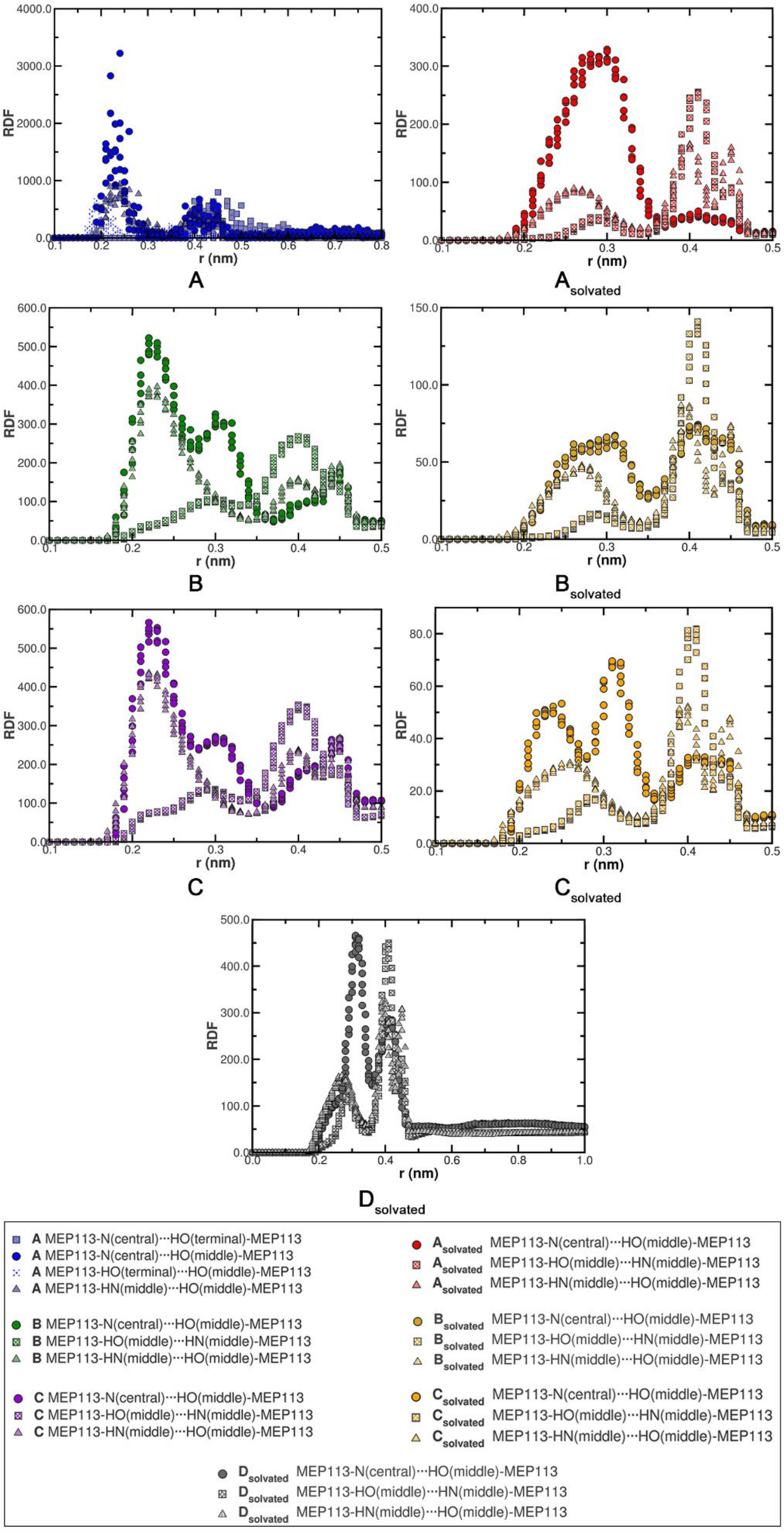

Figure S15. Radial distribution functions (RDFs) between specific atoms of MEP113 units in dry systems (**A**–**C**). These results reveal preferred intramolecular and intermolecular contacts under different conditions.

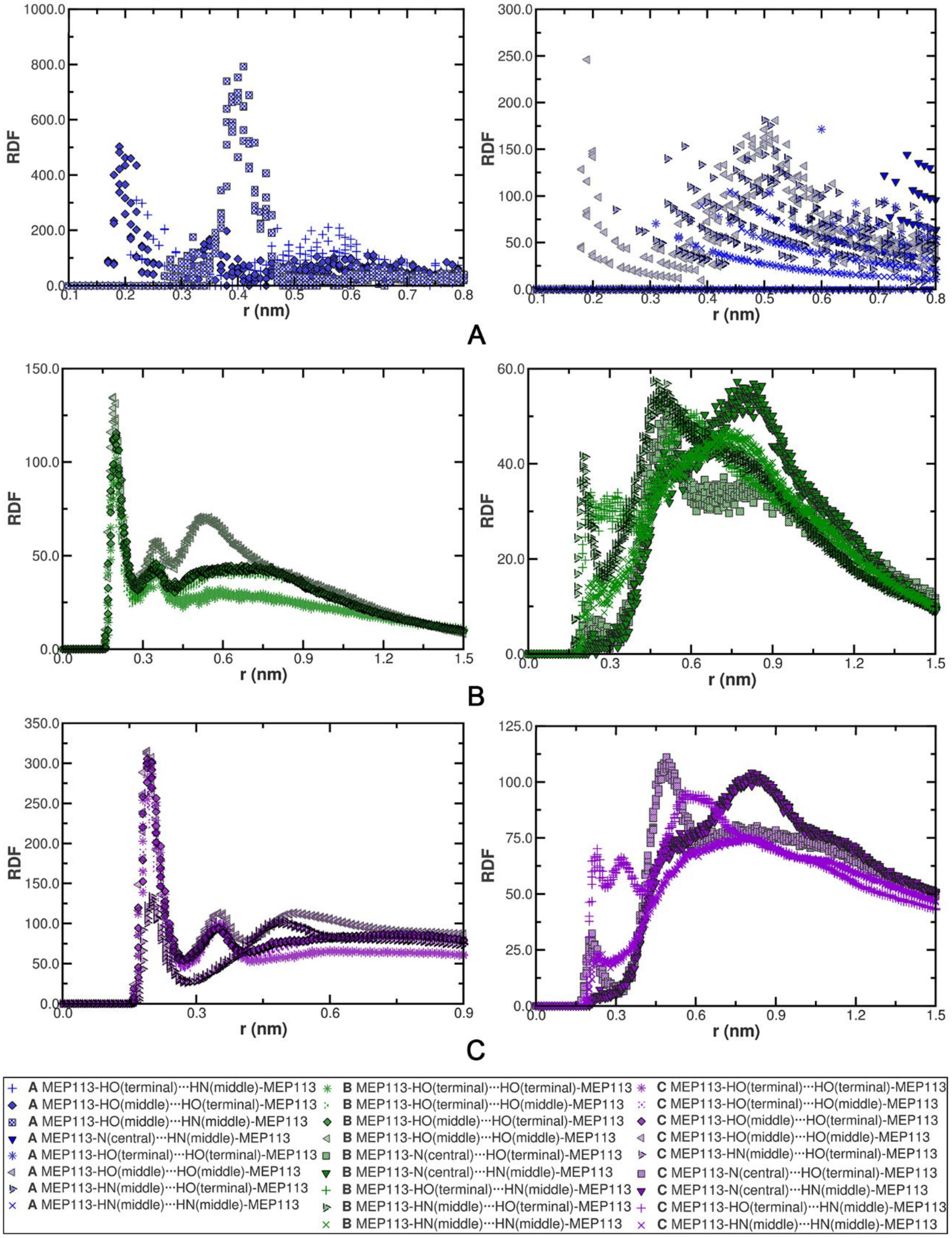

Figure S16. Radial distribution functions (RDFs) between specific atoms of MEP113 units in dry systems (**A**–**C**). These results reveal preferred intramolecular and intermolecular contacts under different conditions.

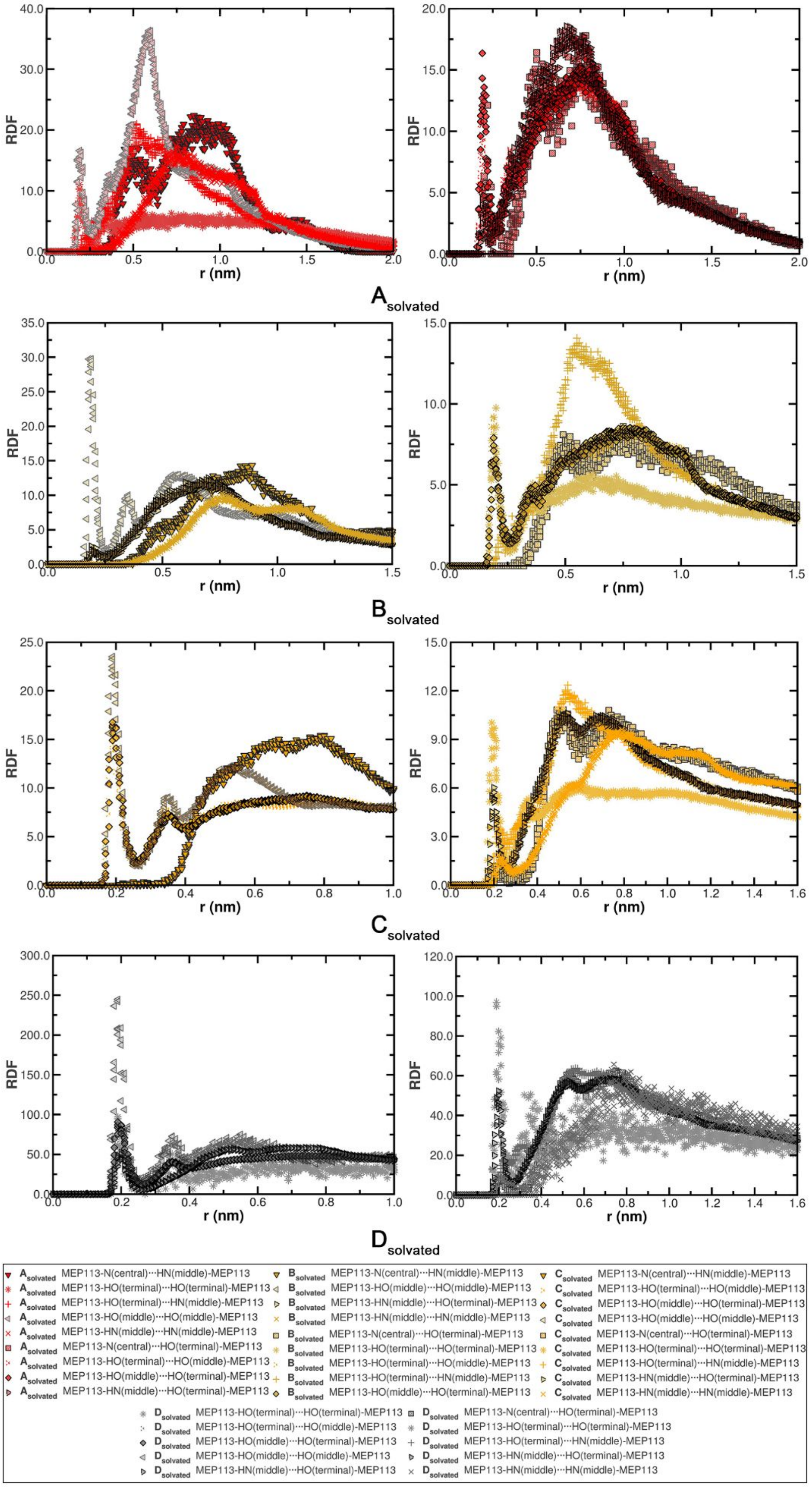

Figure S17. Radial distribution functions (RDFs) between specific atoms of MEP113 units in solvated systems (**A**$_{\mathbf{solvated}}$–**D**$_{\mathbf{solvated}}$). These results reveal preferred intramolecular and intermolecular contacts under different conditions.

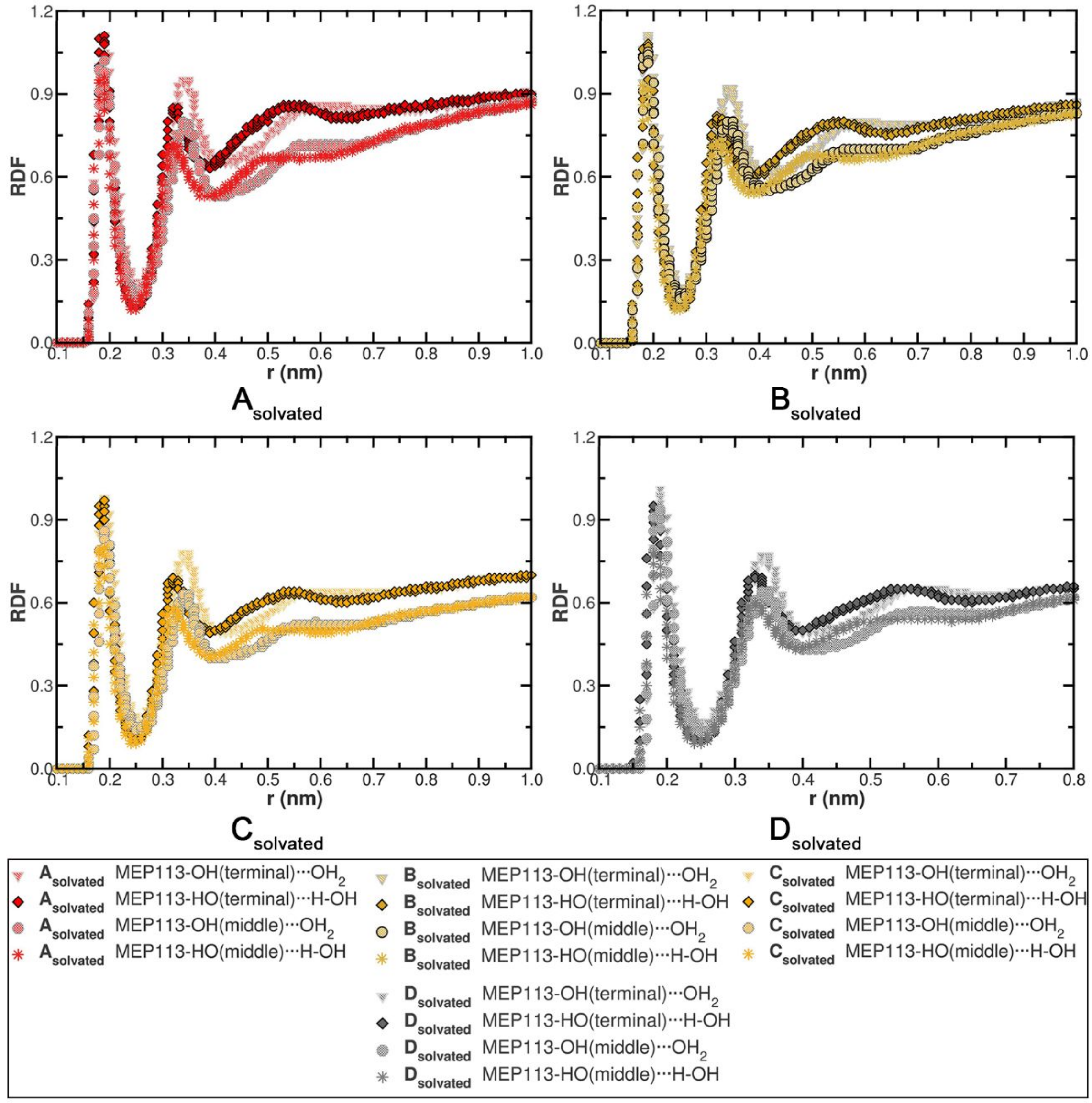

Figure S18. Radial distribution functions (RDFs) of polymer–water atomic pairs in solvated systems, highlighting hydrogen–bonding interactions between hydroxyl/amine groups of MEP113 and surrounding water molecules, key to solvation and stability.

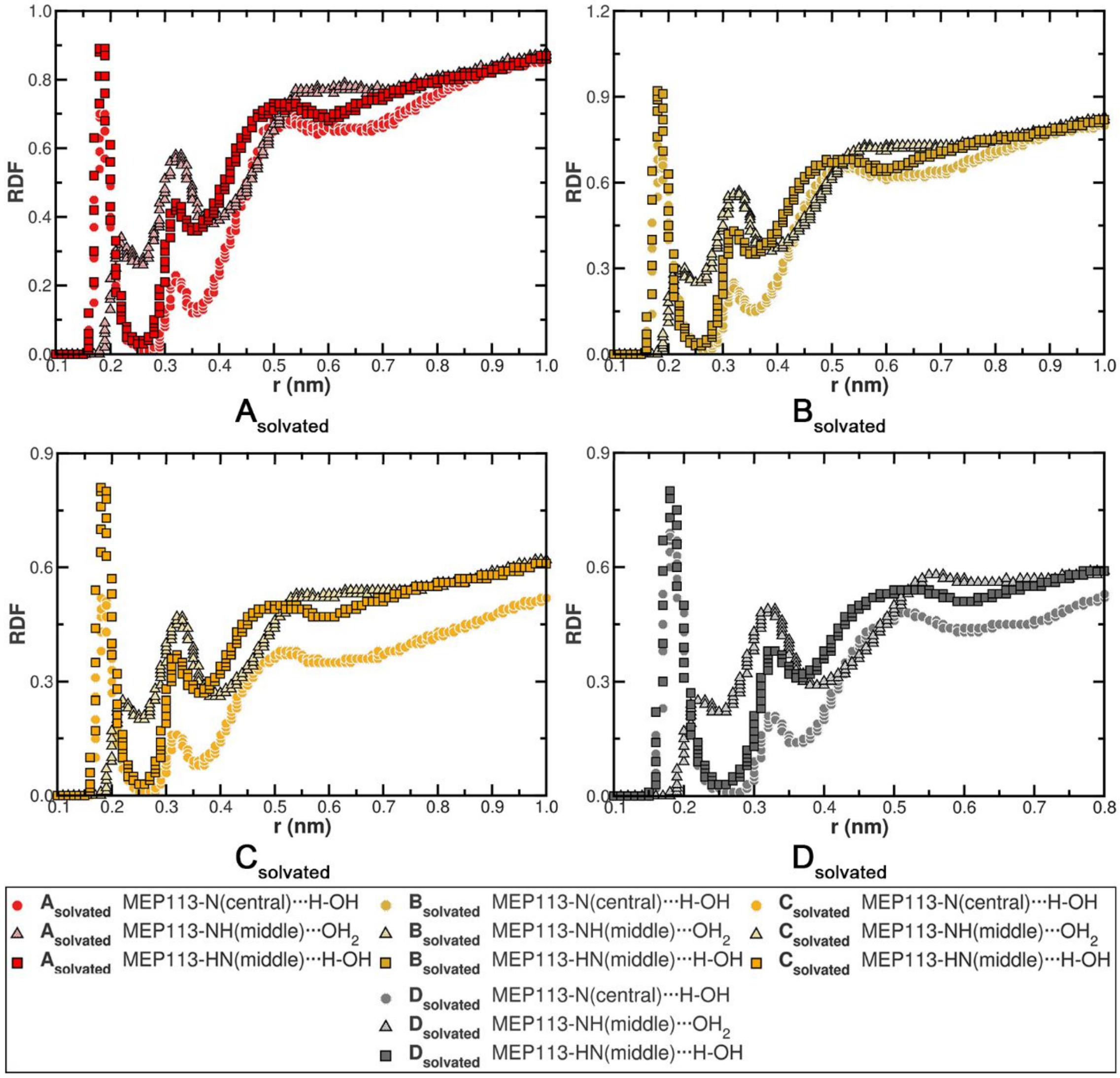

Figure S19. Radial distribution functions (RDFs) of polymer–water atomic pairs in solvated systems, highlighting hydrogen–bonding interactions between hydroxyl/amine groups of MEP113 and surrounding water molecules, key to solvation and stability.

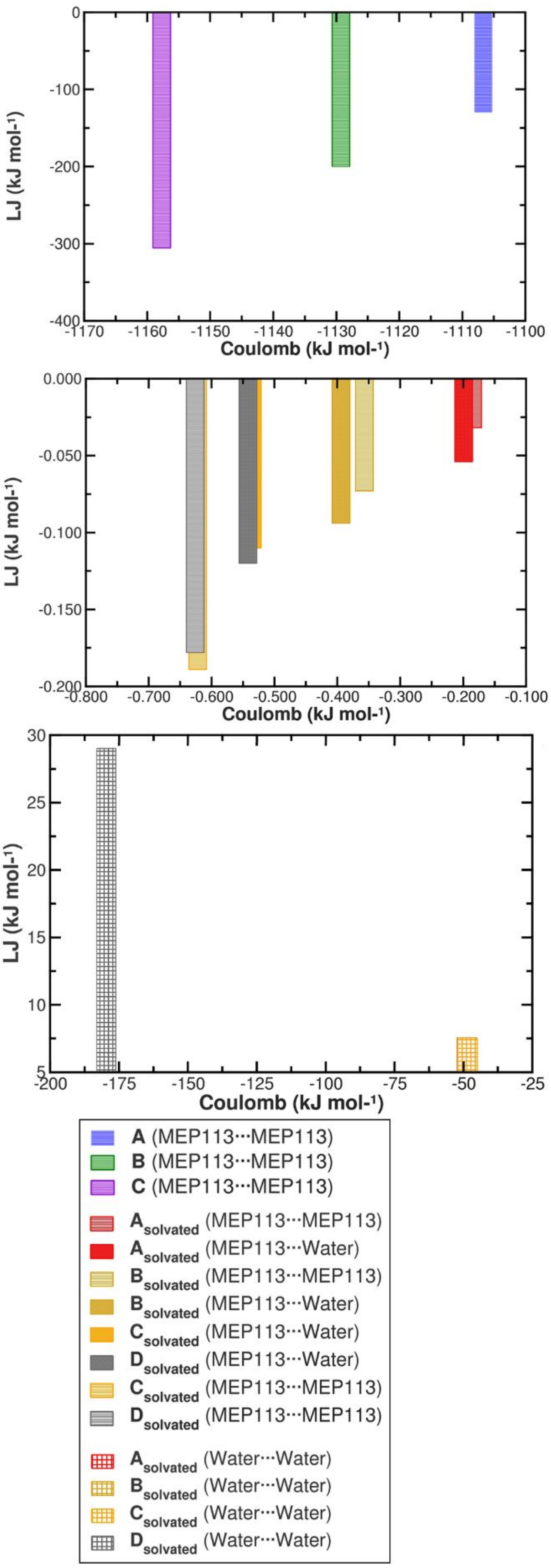

Figure S20. Average Coulomb and Lennard–Jones interaction energies (kJ mol^{-1}) between polymer units and between polymer–water pairs in systems **A**–**C** and **A$_{solvated}$–D$_{solvated}$**, calculated over the last equilibration windows. Normalization was done per polymer unit (dry systems) or per water molecule (solvated systems). The results highlight how solvation modulates electrostatic and dispersive stabilization.

Table S1. Average and standard deviation values of radius of gyration (Rg) and end–to–end distance (nm) for MEP113 in systems **A**–**D**$_{\mathbf{solvated}}$. These parameters quantify molecular flexibility and complement the structural analyses and the NCI–based insights into polymer–water stabilization.

System	Parameter	Average	Standard Deviation
**A**	Rg	1.045	0.140
	End–to–End Distance	2.883	0.426
**B**	Rg	0.793	0.053
	End–to–End Distance	1.400	0.372
**C**	Rg	0.879	0.028
	End–to–End Distance	1.708	0.196
**A**$_{\mathbf{solvated}}$	Rg	0.816	0.103
	End–to–End Distance	1.643	0.541
**B**$_{\mathbf{solvated}}$	Rg	0.922	0.073
	End–to–End Distance	2.077	0.348
**C**$_{\mathbf{solvated}}$	Rg	0.962	0.017
	End–to–End Distance	2.227	0.123
**D**$_{\mathbf{solvated}}$	Rg	1.771	0.089
	End–to–End Distance	0.920	0.026

Repository of Simulation Data

Structures (GRO files) and videos (GIF files) related to the final NVT/NPT equilibration for all investigated systems, as well as all MDP, TOP, and ITP files used in these simulations, are available in the following repository: https://github.com/molmodcs/Polyetheramine-Epoxide-Gels-Molecular-Dynamics.git.
