# Supplementary figures and images for: Solvation-Driven Self-Assembly of Polyetheramine–Epoxide Gels: Insights from Molecular Simulations
Source: ACS Polym Au. 2025 Dec 30;6(1):405–14. doi: 10.1021/acspolymersau.5c00159 (PMC12903512; doi:10.1021/acspolymersau.5c00159)

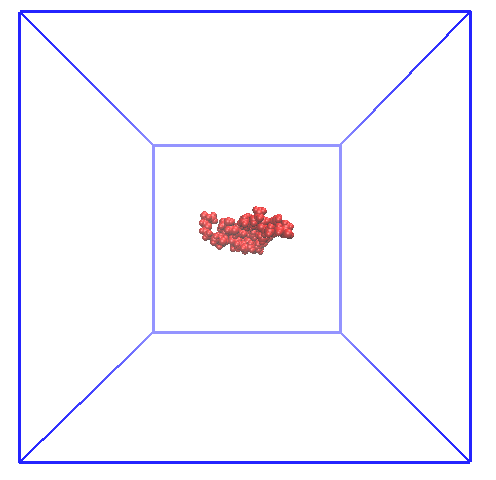

Supplement: Supplementary file 5 [file lg5c00159_si_005.zip › Videos/A.gif]

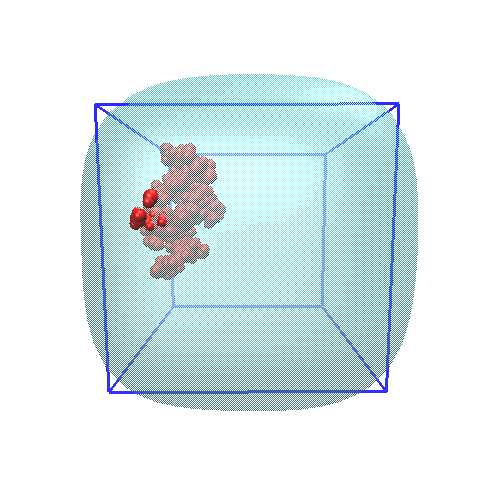

Supplement: Supplementary file 5 [file lg5c00159_si_005.zip › Videos/A_solvated.gif]

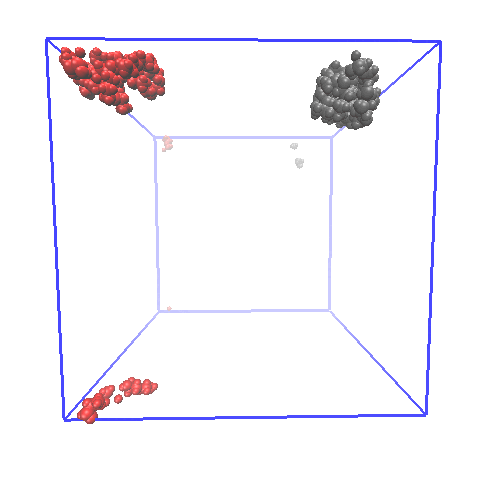

Supplement: Supplementary file 5 [file lg5c00159_si_005.zip › Videos/B.gif]

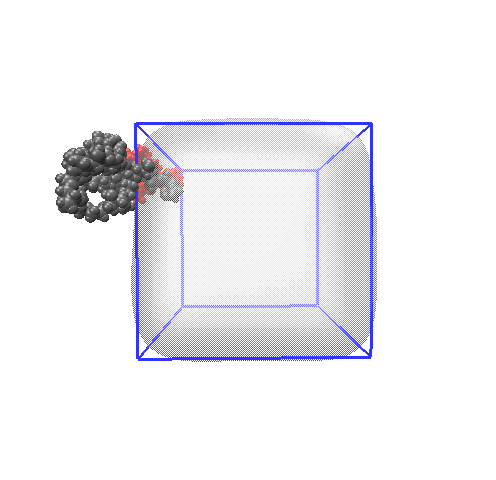

Supplement: Supplementary file 5 [file lg5c00159_si_005.zip › Videos/B_solvated.gif]

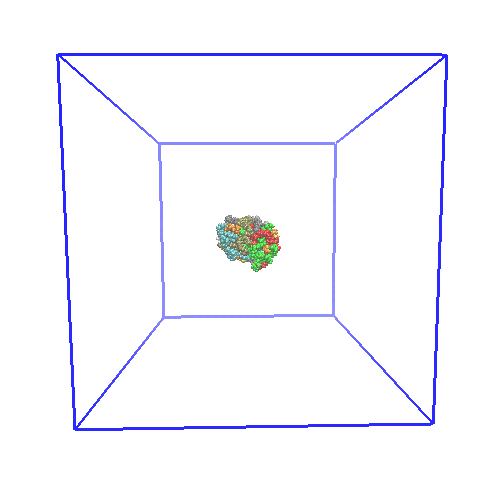

Supplement: Supplementary file 5 [file lg5c00159_si_005.zip › Videos/C.gif]

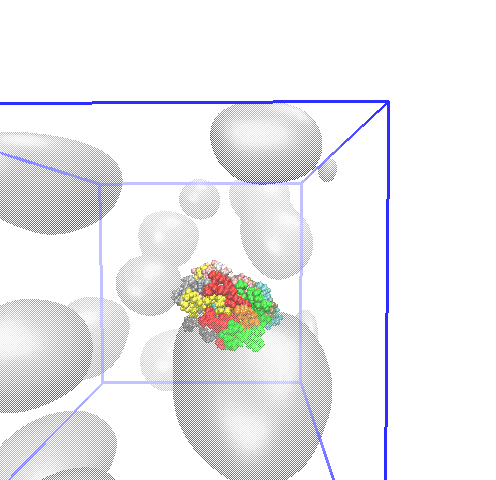

Supplement: Supplementary file 5 [file lg5c00159_si_005.zip › Videos/C_solvated.gif]

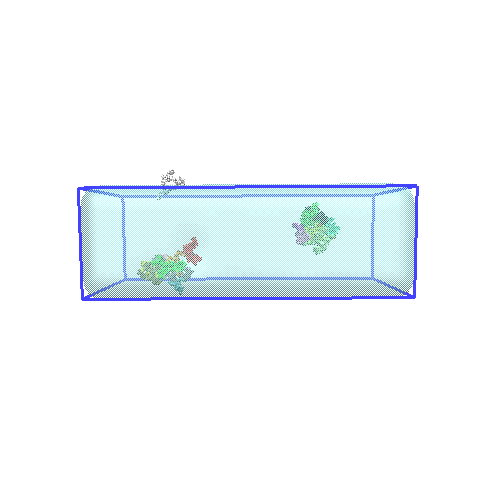

Supplement: Supplementary file 5 [file lg5c00159_si_005.zip › Videos/D_solvated_100ns.gif]
